# Characterization of *Amycolatopsis* 75iv2 dye-decolorizing peroxidase on *O*-glycosides

**DOI:** 10.1128/aem.00205-24

**Published:** 2024-04-16

**Authors:** Silja Välimets, Peicheng Sun, Ludovika Jessica Virginia, Gijs van Erven, Mark G. Sanders, Mirjam A. Kabel, Clemens Peterbauer

**Affiliations:** 1 Laboratory of Food Biotechnology, Department of Food Science and Technology, University of Natural Resources and Life Sciences, Vienna (BOKU), Muthgasse, Vienna, Austria; 2 Doctoral Programme Biomolecular Technology of Proteins (BioToP), BOKU, Muthgasse, Vienna, Austria; 3 Laboratory of Food Chemistry, Wageningen University and Research, Bornse Weilanden, Wageningen, the Netherlands; 4 Wageningen Food and Biobased Research, Wageningen University and Research, Bornse Weilanden, Wageningen, the Netherlands; University of Milano-Bicocca, Milan, Italy

**Keywords:** dye-decolorizing peroxidase, *O*-glycosides, recombinant expression

## Abstract

Dye-decolorizing peroxidases are heme peroxidases with a broad range of substrate specificity. Their physiological function is still largely unknown, but a role in the depolymerization of plant cell wall polymers has been widely proposed. Here, a new expression system for bacterial dye-decolorizing peroxidases as well as the activity with previously unexplored plant molecules are reported. The dye-decolorizing peroxidase from *Amycolatopsis* 75iv2 (DyP2) was heterologously produced in the Gram-positive bacterium *Streptomyces lividans* TK24 in both intracellular and extracellular forms without external heme supplementation. The enzyme was tested on a series of *O*-glycosides, which are plant secondary metabolites with a phenyl glycosidic linkage. *O*-glycosides are of great interest, both for studying the compounds themselves and as potential models for studying specific lignin-carbohydrate complexes. The primary DyP reaction products of salicin, arbutin, fraxin, naringin, rutin, and gossypin were oxidatively coupled oligomers. A cleavage of the glycone moiety upon radical polymerization was observed when using arbutin, fraxin, rutin, and gossypin as substrates. The amount of released glucose from arbutin and fraxin reached 23% and 3% of the total substrate, respectively. The proposed mechanism suggests a destabilization of the ether linkage due to the localization of the radical in the para position. In addition, DyP2 was tested on complex lignocellulosic materials such as wheat straw, spruce, willow, and purified water-soluble lignin fractions, but no remarkable changes in the carbohydrate profile were observed, despite obvious oxidative activity. The exact action of DyP2 on such lignin-carbohydrate complexes therefore remains elusive.

**IMPORTANCE:**

Peroxidases require correct incorporation of the heme cofactor for activity. Heterologous overproduction of peroxidases often results in an inactive enzyme due to insufficient heme synthesis by the host organism. Therefore, peroxidases are incubated with excess heme during or after purification to reconstitute activity. *S. lividans* as a production host can produce fully active peroxidases both intracellularly and extracellularly without the need for heme supplementation. This reduces the number of downstream processing steps and is beneficial for more sustainable production of industrially relevant enzymes. Moreover, this research has extended the scope of dye-decolorizing peroxidase applications by studying naturally relevant plant secondary metabolites and analyzing the formed products. A previously overlooked artifact of radical polymerization leading to the release of the glycosyl moiety was revealed, shedding light on the mechanism of DyP peroxidases. The key aspect is the continuous addition, rather than the more common approach of a single addition, of the cosubstrate, hydrogen peroxide. This continuous addition allows the peroxidase to complete a high number of turnovers without self-oxidation.

## INTRODUCTION

In 1998, new dye-decolorizing enzymes from *Geotrichum candidum* Dec 1 and *Bjerkandera adusta* were discovered, which was the basis of a novel class of peroxidases known as dye-decolorizing peroxidase (DyP) (1, 2). These enzymes do not show relevant sequence homology to classic fungal heme peroxidases such as manganese peroxidases, versatile peroxidases, or lignin peroxidases (3). DyPs are divided into four classes (i.e., A, B, or C/D). Class A covers bacterial enzymes that contain an N-terminal signal sequence and is thus predicted to be secreted. Class B also comprises bacterial enzymes but smaller in size than the ones in class A. In class C/D, both bacterial and fungal enzymes are grouped. This class contains the most active DyPs that are currently known (4, 5).

Overall, DyPs are heme-containing biocatalysts that oxidize a variety of aromatic compounds by using hydrogen peroxide as oxidant. The catalytic cycle of DyPs has been shown to be similar to plant peroxidases and includes an enzyme resting state and the transient intermediates referred to as compound I and compound II (6, 7). Compound I ([Fe^4+^=O]**^+·^**) is formed from the resting state (Fe^3+^) by a heterolytic cleavage of the two-electron equivalent oxidant hydrogen peroxide. A one-electron equivalent substrate reduces compound I to compound II (Fe^4+^=O), and compound II is further reduced to the resting state by a second one-electron equivalent substrate. The oxidized substrates with unpaired electrons are unstable and reactive, resulting in oligomerized products (8–10). Typically, the enzymes are identified by being able to oxidize anthraquinone, azo, indigoid, or xanthene dyes that other peroxidases are poorly active with (3, 11–15). Additionally, the scope of DyP activity was extended to carotenoids (12, 16).

Although the physiological role of these enzymes has yet to be discovered and may be diverse in organisms harboring more than one enzyme, a function in radical-mediated depolymerization of plant biomass is widely accepted (17–19). Among many other bacterial strains, the soil bacterium *Amycolatopsis* 75iv2 and *Bacillus amyloliquefaciens* isolated from cattle feces have been previously reported to modify lignin (20–22). These lignin decomposers encode for DyP2 and *Ba*DyP, which *in vitro* showed activity toward the dimeric phenolic lignin model compound guaiacyl glycerol-β-guaiacyl ether (15, 23, 24). DyP from another soil bacterium, *Bacillus subtilis*, named *Bs*DyP, was shown to degrade the non-phenolic lignin dimer veratryl glycerol-β-guaiacyl ether at the β-*O*-4 linkage (25). Additionally, DyP activity was studied on complex biomass and technical lignin (17, 18, 26, 27).

In order to widen the applicational potential of DyPs, we aimed to investigate a previously unexplored activity on secondary metabolites of plants. *O*-glycosides are composed of an aromatic moiety (aglycone) and an *O*-linked carbohydrate moiety (glycone) that occur naturally throughout the plant kingdom with biological functions as protective agents against damaging radiation or invasion of pathogenic microorganisms (28, 29). Alizarin, an anti-fungal anthraquinone, was shown to be degraded by fungal secreted DyP (30). A plausible physiological role for DyPs is therefore the degradation of such anti-microbials. Moreover, *O*-glycosides present a phenyl glycosidic bond as one of the suggested lignin-carbohydrate complex (LCC) linkages (31). Due to the complexity of the plant polymer matrix and the relatively low abundance of LCCs, *O*-glycosides can be useful model compounds to study DyP activity on phenyl glycoside type LCCs.

Another challenge for DyP characterization is their heterologous production. Most bacterial peroxidases for research purposes were produced in *Escherichia coli* (11, 13, 23, 27, 32–34). Although *E. coli* production systems result in high yields of recombinant intracellular protein, the obtained peroxidases are often inactive due to failed or insufficient incorporation of heme (23, 25, 27). Thus, typically bacterial peroxidase production in *E. coli* requires an additional downstream heme supplementation step. After heme reconstitution, holoenzymes with an incorporated heme cofactor generate a unique ultraviolet-visible (UV-Vis) spectrum with a Soret band at 407–411 nm, and microenvironment changes (i.e*.,* bound imidazole as a common reagent in the protein purification processes) in the porphyrin ring with a ligated iron causes a shift in the Soret band (35), allowing performance of quality control of high-spin Fe^3+^ hemoproteins.

Using the Gram-positive bacterium *Streptomyces lividans* TK24 as an expression host, we established a novel intracellular and extracellular production system for the most active bacterial DyP from *Amycolatopsis* 75iv2, named DyP2. The enzyme activity was assessed on *O*-glycosides such as salicin, arbutin, fraxin, rutin, naringin, and gossypin. All reactions were analyzed using ultraperformance liquid chromatography-mass spectrometry (UHPLC-MS^n^) and high-performance anion-exchange chromatography (HPAEC). The reactions with arbutin and fraxin were further analyzed with matrix-assisted laser desorption/ionization time-of-flight mass spectrometry (MALDI-TOF MS) and high-resolution mass spectrometry (IQ-X). In addition, DyP2 activity on complex lignocellulosic materials as a native source of LCCs such as wheat straw, spruce, willow, and purified water-soluble lignin fractions was analyzed using HPAEC and pyrolysis gas chromatography-mass spectrometry.

## RESULTS

### Heterologous production of DyP2 in *S. lividans*

*Amycolatopsis* 75iv2 C-type DyP, named DyP2, was intracellularly produced in *S. lividans* TK24 (Fig. S1A) because its N-terminal amino acid sequence does not contain characteristics of a putative signal sequence. Subsequently, one-step affinity chromatography yielded approximately 90% pure enzyme. The Soret band maximum was at 429 nm; hence, the protein was dialyzed overnight, resulting in a shift in the Soret band maximum to 410 nm, indicating successful removal of the hindering imidazole (Fig. S1B). The intracellular production of DyP2 resulted in 13 ± 2.8 mg/L of protein with an Reinheitszahl value (*R*_*z*_) (*A*_411 nm_/*A*_280 nm_) of 1.4 ± 0.10, without external heme supplementation.

**Fig 1:**
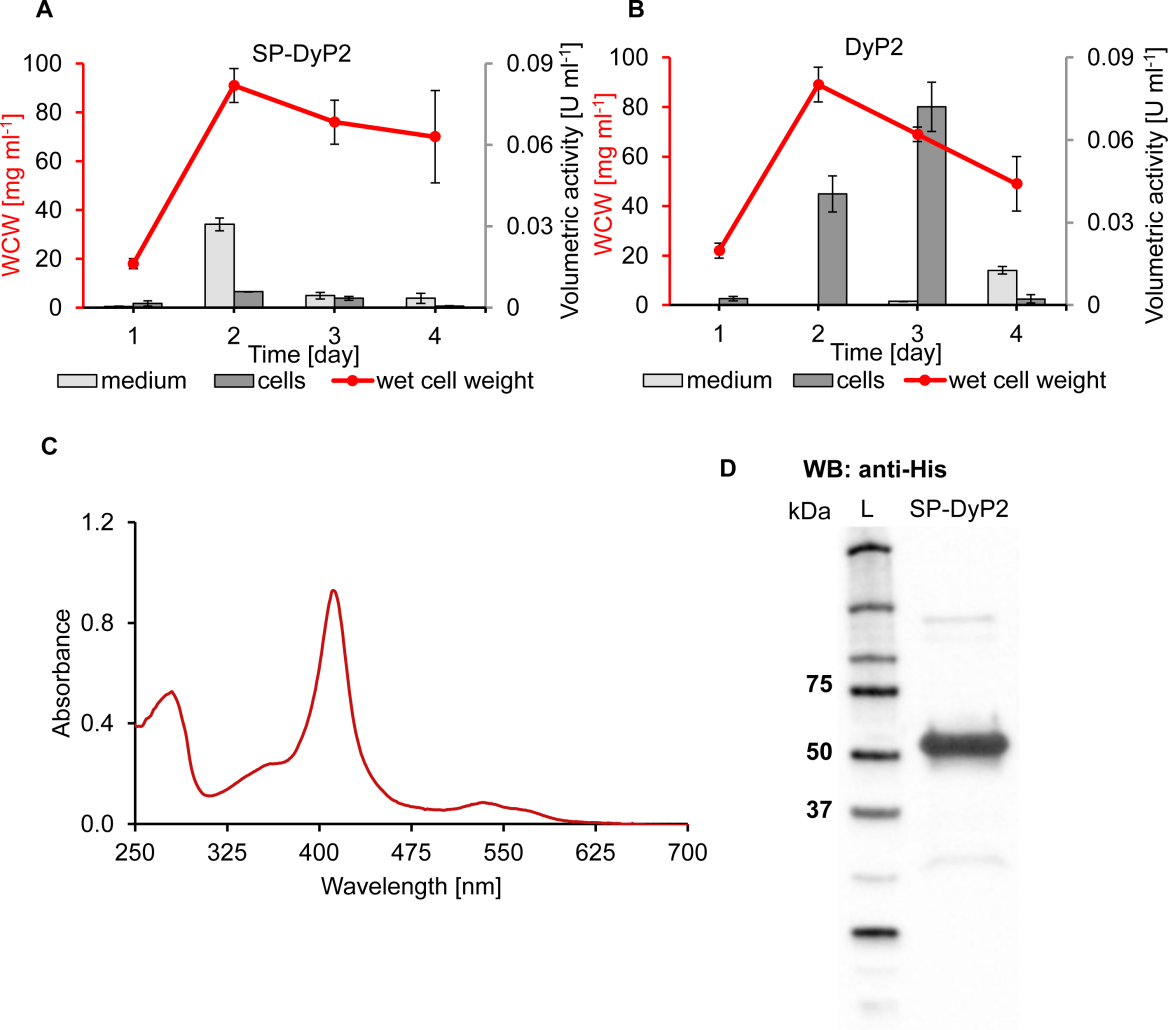
Extracellular production of DyP2 in *Streptomyces lividans*. Cells were cultured in selective growth medium, and samples were taken at defined time points. The supernatant was harvested by centrifugation, and the activity was measured spectrophotometrically. The activity measurements are an average of three biological replicates in three technical replicates each. Wet cell weight (WCW) was measured to follow bacterial growth. (**A**) Secretion of DyP2 using the signal peptide from *B. subtilis* A-type DyP (SP-DyP2). (**B**) Secretion of DyP2 without signal peptide (DyP2). (**C**) Ultraviolet-visible spectrum of secreted and purified DyP2. The enzyme was purified from the culture medium after 2 days of production using affinity chromatography. The maximum absorption of the Soret band was at 411 nm.(**D**) Western blot (WB) analysis of purified SP-DyP2. Sizes 75, 50, and 37 kDa are marked on the ladder (L). The expected size of DyP2 based on theoretical molecular weight calculation after secretion and cleavage of the signal peptide was 52 kDa.

In order to ease downstream processing, the same DyP2 was produced extracellularly. *S. lividans* was again used, because earlier studies have shown promising results of *S. lividans* as expression host for secretory proteins from Actinomycetes (36–38). Because DyP2 belongs to a C-type DyP subfamily, it does not present an N-terminal signal sequence for extracellular production. Therefore, the heterologous signal sequence of *B. subtilis* DyP (*Bs*DyP) (Fig. S1C) was cloned into the pIJ plasmid upstream and in-frame with the coding region of the *DyP2* gene, and the strain expressing this construct was cultured for 4 days. Samples taken once a day were subjected to enzyme activity measurements in the culture supernatant and in the cell extracts using the 2,2′-azino-bis(3-ethylbenzothiazoline-6-sulfonic acid) (ABTS) assay. Due to the heterogeneous mycelial growth of *S. lividans*, the wet cell weight was used to monitor viability (36, 39, 40). The cells producing DyP2 with the *Bs*DyP signal peptide (SP-DyP2) reached their maximal biomass on day 2 (Fig. 1A). SP-DyP2 activity in the medium reached a maximum of 0.03 U/mL on day 2, whereas the activity in the cell extracts was significantly lower. This suggested that most of the produced enzyme was secreted. SP-DyP2 wet cell weight started to decrease on day 3, suggesting the onset of cell lysis. SP-DyP2 activity was significantly decreased on days 3 and 4, suggesting degradation of the produced enzyme. The native *DyP2*-encoding gene without sequence for a signal peptide (DyP2) was used as a non-secretion control (Fig. 1B). The DyP2 strain also reached the highest wet cell weight on day 2, and enzyme activity could be measured only in the cell extracts, indicating that no enzyme was secreted into the medium. On day 3, DyP2 activity in the cell extracts reached its maximum but decreased rapidly on day 4, again suggesting degradation. DyP2 activity could be measured in the culture medium on day 4, concomitant with a decrease in wet cell weight after reaching a peak on day 2.

To verify that the N-terminal amino acid sequence was properly processed during secretion, SP-DyP2 was purified from the culture supernatant using affinity chromatography. The enzyme showed a heme protein-like spectrum with a Soret band maximal absorption at 411 nm (Fig. 1C) resulting in an *R*_*z*_ (*A*_411 nm_/*A*_280 nm_) value of 1.75 without external heme addition. Anti-his Western blot analysis showed a band around 52 kDa size, confirming the successful production of full-length DyP2 (Fig. 1D). The cleavage of the *Bs*DyP signal peptide was verified by N-terminal sequencing (data not shown), resulting in mature DyP2.

The possibility to produce active extracellular heterologous DyP is shown, but purified extracellular protein yields remained lower compared to intracellular production (data not shown). The optimization of the DyP secretion was out of scope for the current research, and further experiments were performed with intracellularly produced DyP2.

### Characterization of DyP2 on *O*-glycosides

**Fig 2:**
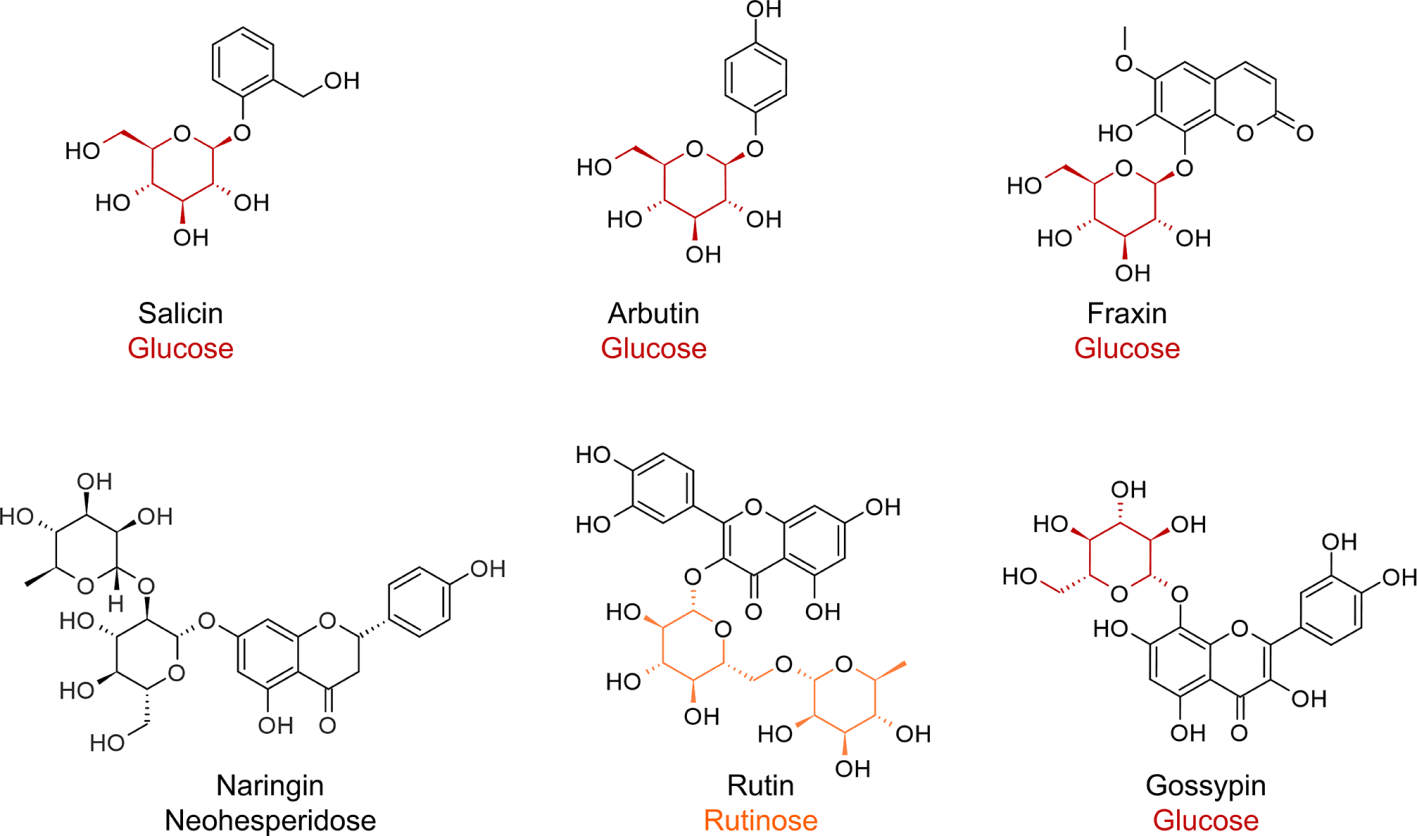
Overview of *O*-glycosides used in this study. Salicin, arbutin, and fraxin containing ether-linked glucosyl moiety were fully soluble in reaction buffer. Naringin, rutin, and gossypin containing ether-linked neohesperidosyl, rutinosyl, or glucosyl moieties, respectively, were partially soluble in reaction buffer.

**Fig 3:**
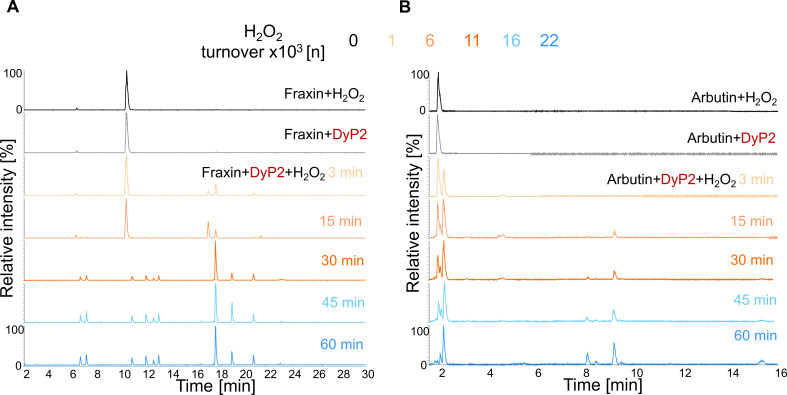
UHPLC-MS time course chromatograms in negative mode. (**A**) Fraxin reaction products and (**B**) arbutin reaction products. Substrates with a final concentration of 2.6 mM were solubilized in 50 mM sodium acetate buffer at pH 4.5; 0.2 µM enzyme was added, and the reaction was initiated with 200 µM H_2_O_2_. The reaction was continued by adding fresh H_2_O_2_ after every 3 min for 60 min. The H_2_O_2_ turnover per time point is color coded above the figures. Samples were taken at 3, 15, 30, 45, and 60 min. Substrates containing only H_2_O_2_ or DyP2 were used as controls. The incubations were performed in duplicate, but only one of the identical replicates is shown. Tentatively annotated products of fraxin and arbutin incubations are listed in Table 1 or 2, respectively.

Several *O*-glycosides (Fig. 2), selected based on differences in aromatic backbone, number of phenolic groups, and various carbohydrate moieties,were used to study DyP2 activity. Salicin, arbutin, fraxin, and gossypin have ether-linked glucosyl moieties, whereas naringin and rutin have ether-linked neohesperidosyl and rutinosyl moieties, respectively (Fig. 2). Salicin, arbutin, and fraxin were soluble in sodium acetate buffer (pH 4.5) at the concentrations used (2.6 mM). The more complex glycosides, naringin, rutin, and gossypin, were only partially soluble in the reaction buffer. DyP2 was added to the substrates, and the reaction was initiated with H_2_O_2_. The reaction was continued by adding fresh H_2_O_2_ after every 3 min for 60 min. The conversion of H_2_O_2_ is quantified in terms of how many turnovers of H_2_O_2_ the enzyme performed, which is calculated by dividing the concentration of supplemented H_2_O_2_ with the concentration of the enzyme. Samples were subjected to UHPLC-MS (Fig. 3; Fig. S2 through S5).

**TABLE 1:** Compounds detected with UHPLC-MS after incubation of fraxin with DyP2^*a*^^,*b*^

RT	Tentative annotation	UV_max_ (nm)	Ion	*m*/*z*	RA
3.0	Unknown	204	[M-H]^−^	741	100
6.33	Unknown	336	[M-H]^−^	339	100
6.63	Dimer	228	[M-H]^−^	737	100
7.13	Dimer	228	[M-H]^−^	737	100
8.37	Unknown	204	[M-H]^−^	531	100
10.42	Fraxin	332	[M-H]^−^	369	100
10.87	Oligomer-glucose	232	[M-H]^2−^	552	100
10.87	Oligomer-glucose	232	[M-H]^−^	1,105	85
11.25	Unknown	228	[M-H]^−^	531	100
11.99	Oligomer-glucose	228	[M-H]^2−^	552	100
11.99	Oligomer-glucose	228	[M-H]^−^	1,105	50
12.63	Oligomer-glucose	228	[M-H]^−^	1,105	100
13.05	Oligomer-glucose	228	[M-H]^−^	1,105	100
16.52	Unknown	228	[M-H]^2−^	1,100	100
17.11	Dimer	232	[M-H]^−^	737	100
17.72	Dimer	380	[M-H]^−^	737	100
19.06	Unknown	228	[M-H]^−^	709	100
20.81	Oligomer-glucose	232	[M-H]^2−^	552	100
20.81	Oligomer-glucose	232	[M-H]^−^	1,105	14
21.44	Oligomer-glucose	232	[M-H]^−^	1,105	100
23.02	Unknown	228	[M-H]^2−^	736	100

^*a*^ *m*/*z*, mass-to-charge ratio; RA, relative abundance; RT, retention time.

^*b*^ Full table with fragmentation info is in the Table S1.

**TABLE 2:** Compounds detected with UHPLC-MS after incubation of arbutin with DyP2^*b*^

RT	Tentative annotation	UV_max_ (nm)	Ion	*m*/*z*	RA^*a*^
1.87	Arbutin	280	[M + HCOO^−^]^−^	317	100
1.97	Trimer	232	[M-H]^−^	811	100
2.12	Dimer	232	[M-H]^−^	541	100
2.12	Unknown	232	[M-H]^−^	1,083	26
3.07	Trimer	208	[M-H]^−^	811	100
3.07	Unknown	208	[M-H]^−^	1,081	32
4.45	Trimer	228	[M-H]^−^	811	100
4.59	Dimer	228	[M + HCOO^−^]^−^	587	100
4.59	Dimer	228	[M-H]^−^	541	63
5.47	Unknown	208	[M-H]^−^	377	100
5.47	Unknown	228	[M-H]^−^	1,125	64
8.11	Trimer-glucose	228	[M-H]^−^	647	100
8.47	Unknown	228	[M-H]^−^	917	100
8.94	Trimer	228	[M + HCOO^−^]^−^	857	100
8.94	Trimer	228	[M-H]^−^	811	85
9.21	Trimer-glucose	228	[M-H]^−^	647	100
9.49	Unknown	228	[M-H]^−^	917	100
9.49	Unknown	228	[M + HCOO^−^]^−^	963	45
11.56	Unknown	228	[M-H]^−^	917	100
15.36	Trimer-glucose	228	[M + HCOO^−^]^−^	693	100
15.36	Trimer-glucose	228	[M-H]^−^	647	40

^*a*^ RA, relative abundance.

^*b*^ Full table with fragmentation info is in the Table S2.

Notably, the color of the all the reactions with compounds presented in Fig. 2 changed significantly after 60 min or after 22,000 turnovers of H_2_O_2_. Arbutin was colorless and obtained a brown color after incubation with DyP2 (Fig. S6A). The partially soluble substrates rutin and gossypin turned brown after enzymatic treatment (Fig. S6B and C), and naringin obtained a light-yellow color (Fig. S6D). The most interesting color change was observed with fraxin, where the starting compound was colorless, then changed to yellow, followed by an unstable purple intermediate, and finally changed to olive green with concomitant precipitation (Fig. S6E and F). Unfortunately, the intermediate products exhibiting blue reaction with fraxin were difficult to analyze as they were found to be stable for less than an hour. The blue intermediate reaction mixture exhibited completely different UV-Vis spectrum with peaks at 357 and 575 nm compared to the control and 3 min time point (Fig. S6G)

DyP2 showed no conversion of substrate or formation of oligomerized products with salicin (Fig. S7). Fraxin was completely converted after 30 min or after 11,000 turnovers of H_2_O_2_ (Fig. 3A), whereas residual arbutin was still present after 60 min or after 22,000 turnovers of H_2_O_2_ (Fig. 3B). Oligomers, mostly dimers and trimers, were the primary reaction products of all glycosides tested (Table 1; Tables S1 through S5). Besides the major coupling reaction, strikingly, mass-to-charge ratios (*m*/*z*) of fraxin (*m*/*z* 552) (Table 1), arbutin (*m*/*z* 647) (Table 2), and rutin (*m*/*z* 912) (Table S3) indicated the loss of sugar moiety (glucose or rutinose) in the reactions. Note that glycosyl moiety loss in these reactions was detected only from already oligomerized products. For example, the arbutin trimer *m*/*z* 811 presumably had lost one glucosyl moiety, resulting in *m/z* of 647. Still, a number of compounds, especially resulting from the reaction with gossypin, with unfamiliar mass-to-charge ratios, could not be annotated. The UV chromatograms of rutin and naringin (Fig. S3A and S4A) showed a hill-shaped peak indicating an inseparable mixture of heterogenous products. Only a few compounds in this peak were ionized and detected (Tables S3 and S4).

The time and H_2_O_2_ turnover-dependent formation and deformation of compounds in the reactions with fraxin and arbutin are shown in Fig. S8 and S9, respectively. Similar trends in arbutin and fraxin reactions were initial substrate consumption, followed by a formation of oligomerized products (dimers and trimers), and the formation of oligomerized products with presumed loss of the glucosyl moiety. Notably, the formation of dimers and trimers happened earlier (maximum abundance reached at 15 or 30 min) than the formation of oligomers with the loss of glycosyl moiety (maximum abundance reached at 45 or 60 min). Eventually, all oligomeric products decreased in abundance, suggesting that they accumulated as insoluble products with a higher degree of polymerization.

In all reactions with glycosides, several compounds with the same *m*/*z* but different retention times (RTs) were detected, for example, fraxin dimers eluted at 6.63, 7.13, 17.11, and 17.72 min (Table 1); arbutin dimers eluted at 2.12 and 4.59 min; trimers at 1.97, 3.07, 4.45, and 8.94 min (Table 2); rutin dimers eluted at 13.96, 14.43, and 14.97 min (Table S3); and gossypin products with the same *m*/*z* eluted at 18.21 and 21.75 min (Table S5). To explain the retention time differences between the compounds with the same *m*/*z*, arbutin products were analyzed by high-resolution mass spectrometry.

### In-depth analysis of arbutin products with high-resolution mass spectrometry

Due to the complexity of fraxin, rutin, naringin, or gossypin reaction products, an in-depth fragmentation analysis using high-resolution mass spectrometry (MS) with orbitrap detection (IQ-X) was performed only on the reaction products of arbutin after 15-min incubation. Note that the liquid chromatography gradient for the IQ-X experiments was changed starting from 1% eluent B instead of 5% eluent B to ensure better retention on the column. Thus, the identical masses of arbutin dimers at 2.12 and 4.59 min in UHPLC-MS correspond to the dimers eluting at 6.11 and 10.61 min, respectively, in the IQ-X.

To find suitable energies in higher-energy collisional dissociation (HCD), the sample was fragmented using 15%, 25%, and 35% collision energy (Fig. S10 and S11). The arbutin dimer that eluted earlier did not fragment fully even at the highest collisional energy (35%), whereas the later-eluting arbutin dimer was close to complete fragmentation at the lowest energy (15%). Bond dissociation energies were predicted using ALFABET—A machine-Learning derived, Fast, Accurate Bond dissociation Enthalpy Tool (https://bde.ml.nrel.gov/) (41). For dimers with C-C bonds, the predicted energy was 120.7 kcal/mol, and for dimers with C-O linkage, the predicted energy was 74.4–85.6 kcal/mol (Fig. S10).

**Fig 4:**
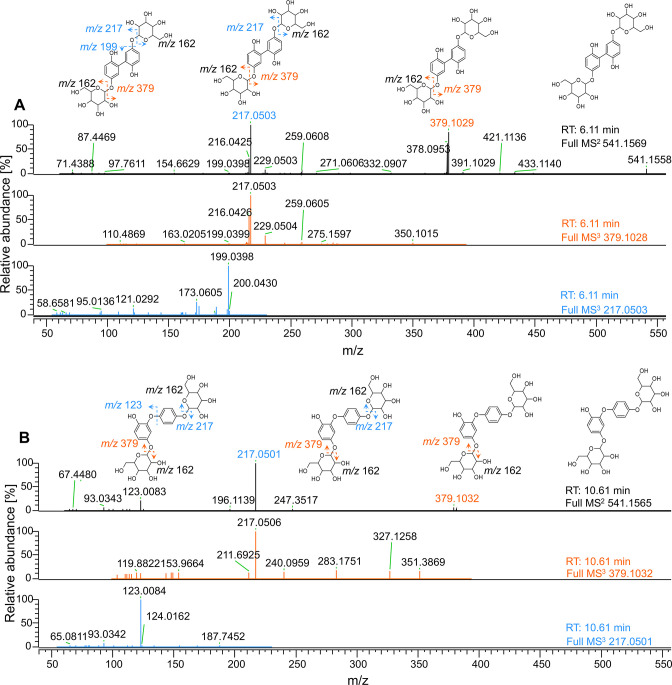
High-resolution fragmentation of arbutin dimers. Arbutin 15-min incubation time point was analyzed in high-resolution MS IQ-X. By using acetonitrile gradient starting from 1%, the dimers eluted at RTs of 6.11 and 10.61 min corresponded to the dimers at RTs 2.12 and 4.59 min in the UHPLC-MS analysis starting at 5% acetonitrile. Full MS^2^ was obtained using HCD 35% collision energy, and obtained MS^2^ fragments were further fragmented in MS^3^ using CID with 35% collision energy. Dimers at RTs of (**A**) 6.11 min and (**B**) 10.61 min have significantly different fragmentation patterns indicating different linkages upon oxidative coupling.

Next, 35% HCD energy was used to determine arbutin dimers and trimers in tandem mass spectrometry (MS^2^). Furthermore, MS^3^ was obtained from precursor MS^2^ ions using 35% ion trap-based collusion-induced dissociation (CID) fragmentation energy (Fig. 4; Fig. S12). The obtained fragments were used to investigate the possible linkage between the arbutin units. The MS^2^ of the dimer with an RT of 6.11 min showed three most prominent fragments: unfragmented dimer (*m*/*z* 541), fragment with the loss of one glucosyl moiety (*m*/*z* 379), and fragment with the loss of two glycosyl moieties (*m*/*z* 217) (Fig. 4A). MS^2^ of the dimer with an RT of 10.61 min showed two most prominent fragments: fragment with the loss of two glucosyl moieties (*m*/*z* 217) and hypothetical aromatic aglycone backbone (*m*/*z* 123) (Fig. 4B). When further fragmenting *m*/*z* 217 for both dimers in MS^3^, the dimer with an RT of 6.11 min showed the most abundance with *m*/*z* 199 and many smaller masses, whereas the dimer with an RT of 10.61 min showed an *m*/*z* of 123. The comparison between theoretical and measured masses is shown in Table S6; the small difference indicates high accuracy of measured masses.

### Formation of larger *O*-glycoside oligomers

Reaction products from conversions of arbutin and fraxin were subjected to MALDI-TOF MS analysis to investigate further polymerization products. The reaction products of arbutin were completely soluble in buffer, so the samples were analyzed as they were. The reaction products of fraxin incubations started to precipitate after 15 min or after 6,000 turnovers of H_2_O_2_ (Fig. S6F) by forming a yellow precipitate. This precipitate was solubilized in methanol/dimethyl sulfoxide (DMSO) mixture and analyzed by MALDI-TOF MS. Measurements were recorded in positive mode, resulting in oligomerization with sodium ion adducts (Tables 3 and 4).

**Fig 5:**
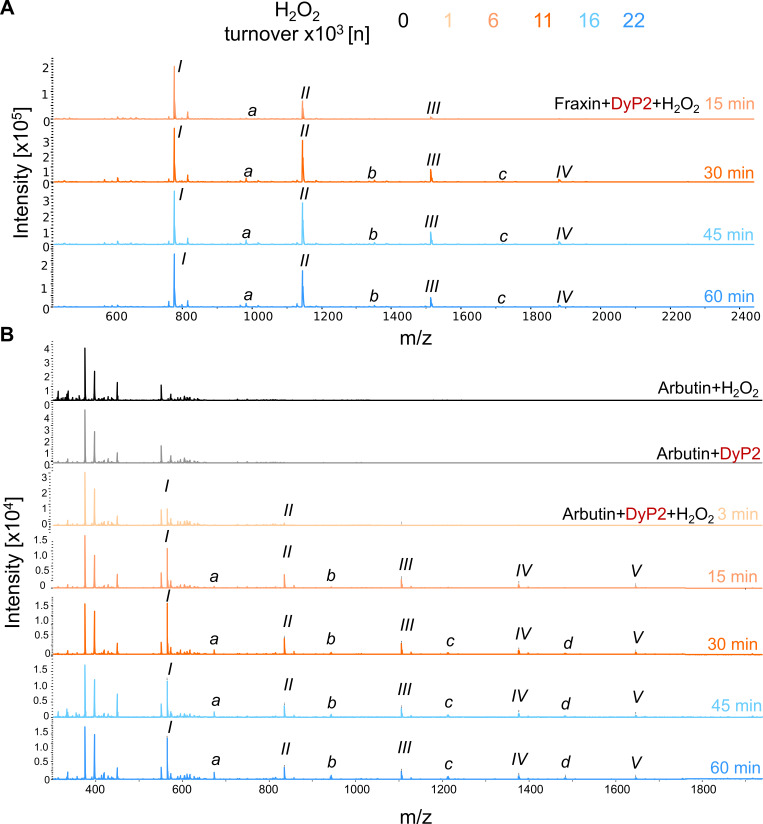
MALDI-TOF MS time course spectra of fraxin and arbutin. Substrates with a final concentration of 2.6 mM were solubilized in 50-mM sodium acetate buffer at pH 4.5; 0.2 µM enzyme was added; and the reaction was initiated with 200 µM H_2_O_2_. The reaction was continued by adding fresh H_2_O_2_ after every 3 min for 60 min. The H_2_O_2_ turnovers per time point are color coded above the figures. Samples were taken at 3, 15, 30, 45, and 60 min. Substrates containing only H_2_O_2_ or DyP2 were used as controls. The incubations were performed in duplicate, but only one of the identical replicates is shown. MALDI-TOF MS was measured in positive mode. (**A**) Fraxin incubation products were partially soluble in buffer. No precipitate was observed after 3 min. The insoluble fractions were observed after 15 min and were separated by centrifugation. The remaining pellet was solubilized in MeOH/DMSO mixture and analyzed. The products are listed in Table 3. (**B**) Arbutin incubation products were fully soluble in buffer. The products are listed in Table 4 as Na^+^ adducts.

Figure 5A shows the time course of fraxin precipitation products. After 15 min or 6,000 turnovers of H_2_O_2_, dimers, trimers, and tetramers were detected. After 30 min, pentamers were also detected. Figure 5B shows the time course of arbutin conversions. After 3 min or 1,000 turnovers of H_2_O_2_, only dimers and trimers were detected (in agreement with the UHPLC-MS results, Table 2), whereas after 15 min or 6,000 turnovers of H_2_O_2_, tetramers, pentamers, and hexamers were detected. The highest degrees of polymerization for arbutin and fraxin were 6 and 5, respectively. It is interesting to note that putative larger oligomers with the loss of a glucosyl unit (marked with *a*, *b*, *c*, and *d*) were again detected in both conversions (Fig. 5; Tables 3 and 4).

**TABLE 3:** Compounds detected with MALDI-TOF MS after fraxin incubation with DyP2

Peak	Tentative annotation	Ion	m/z
I	Fraxin dimer	[M + Na]^+^	777.2
a	Fraxin trimer-glucose	[M + Na]^+^	983.1
II	Fraxin trimer	[M + Na]^+^	1,145.2
b	Fraxin tetramer-glucose	[M + Na]^+^	1,351.2
III	Fraxin tetramer	[M + Na]^+^	1,513.3
c	Fraxin pentamer-glucose	[M + Na]^+^	1,719.3
IV	Fraxin pentamer	[M + Na]^+^	1,881.4

**TABLE 4:** Compounds detected with MALDI-TOF MS after arbutin incubation with DyP2

Peak	Tentative annotation	Ion	*m*/*z*
I	Arbutin dimer	[M + Na]^+^	565.2
a	Arbutin trimer-glucose	[M + Na]^+^	673.3
II	Arbutin trimer	[M + Na]^+^	835.3
b	Arbutin tetramer-glucose	[M + Na]^+^	943.4
III	Arbutin tetramer	[M + Na]^+^	1,105.4
c	Arbutin pentamer-glucose	[M + Na]^+^	1,211.5
IV	Arbutin pentamer	[M + Na]^+^	1,375.5
d	Arbutin hexamer-glucose	[M + Na]^+^	1,483.6
V	Arbutin hexamer	[M + Na]^+^	1,645.6

### Analysis of released glycone moieties in *O*-glycoside reactions

**Fig 6:**
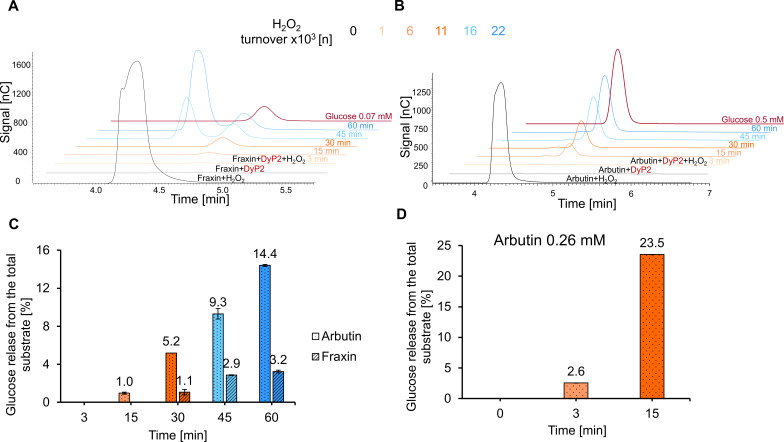
Carbohydrate analysis of *O*-glycoside incubations with DyP2. HPAEC time course chromatograms of released glucose after (**A**) fraxin and (**B**) arbutin incubations. Substrates with H_2_O_2_ or DyP2 only were used as controls. Glucose with defined concentrations were used as standards. The peak eluting at 4.3 min corresponds to unconsumed H_2_O_2_, and the peak eluting at 4.6 min corresponds to glucose. The incubations were performed in duplicate, but only one of the identical replicates is shown. (**C**) Quantification of glucose release from the total substrate. The initial substrate concentration (2.6 mM) was set to 100%. Glucose release was time dependent in both substrate incubations, reaching 14.4% in arbutin and 3.2% in fraxin incubations after 60 min. The average of duplicates is presented. (**D**) Quantification of glucose release from 0.26 mM arbutin. The initial substrate concentration 2.6 mM was decreased 10 times to 0.26 mM and was set to 100%. After the total substrate consumption in 15 min, the glucose yield was 23.5%. The average of duplicates is presented.

UHPLC-MS and MALDI-TOF MS analyses both detected masses indicative of a loss of the carbohydrate moiety in the *O*-glycoside molecule upon radical coupling. HPAEC analysis was performed on the soluble fraction of arbutin, fraxin, rutin, and gossypin conversions to verify the release of glycone in the reaction products with DyP2. No carbohydrates were detected in the controls, confirming that substrates were stable during the reaction conditions used. The peak at 4.3 min in the substrate + H_2_O_2_ control samples corresponded to H_2_O_2_, and the peak at 4.6 min in enzymatically treated samples corresponded to glucose (Fig. S13). Indeed, as shown in Fig. 6A and B and in Fig. S14A and B, glycones were released from arbutin, fraxin, rutin, and gossypin solution.

The release of glucose in *O*-glycoside conversions was dependent on time and substrate (Fig. 6A and B), given the obvious difference between arbutin and fraxin. Fraxin was completely converted after 30 min according to UHPLC-MS analysis. Thus, the additionally added H_2_O_2_ was not consumed but accumulated in the reaction mixture (Fig. 6A), resulting in a peak at 4.3 min. Quantification of free glucose in the conversion from the total substrate (2.6 mM) in fraxin and arbutin conversions was 3% and 14%, respectively. However, UHPLC-MS analysis of arbutin (Fig. 3B) reaction products showed residual substrate after 60 min, indicating incomplete conversion. Thus, to quantify the total amount of sugar release in arbutin, conversions with 0.26 mM arbutin (10 times lower concentration than in the initial experiments) were performed. After 15 min, arbutin was completely consumed, yielding 23% of glucose of the total substrate (Fig. 6D). H_2_O_2_ accumulated after 30 min, indicating that it was not consumed by DyP2 anymore (Fig. S15), thus further indicating absence of the suitable substrate for donating electrons to complete full peroxidase reaction.

To determine whether the sugar release was specific to dye-decolorizing peroxidases or applied to other peroxidases as well, arbutin was incubated with horse radish peroxidase (HRP), which is a plant peroxidase. Figure S16 shows that glucose was detected in the solution after 60 min or after 22,000 turnovers of H_2_O_2_, indicating that glucose was released during HRP-mediated radical coupling.

### Analysis of lignocellulosic substrate upon DyP2 incubation

The observation of the released glycone moieties from arbutin, fraxin, rutin, and gossypin upon reaction with DyP2 raised the question of whether DyP2 induces changes in the carbohydrate profile when tested on complex lignocellulosic material. Hereto, finely milled lignocellulosic materials such as wheat straw, spruce, willow, and water-extractable oligomeric lignin fractions S2 and S4 (42) were incubated with DyP2 to investigate changes in carbohydrate profile. No changes in the carbohydrate content were observed in wheat straw conversion (Fig. S17A). A new peak at 2.5 min was observed in conversions of spruce, willow, and S2 and S4 substrates (Fig. S17B through E). The H_2_O_2_ peak at 4.3 min was not present in enzymatically treated samples, indicating complete consumption of H_2_O_2_ by DyP2 and DyP2 activities on unknown compounds. In addition, a color change accompanied by the formation of precipitate was observed in the samples of spruce, S2, and S4 (Fig. S18).

To further corroborate the DyP activity on complex lignocellulosic substrates, the enzymatically treated water-soluble lignin fractions were analyzed by pyrolysis-gas chromatography (GC)-MS and compared to the control samples. Excitingly, the treated substrates showed a general increase in Cα-oxidized pyrolysis products (Table S7), clearly highlighting oxidative activity. More specifically, aldehyde, ketone, and diketone pyrolysis products all increased, suggesting that various oxidized substructures were formed and that, accordingly, several underlying oxidation pathways occurred.

## DISCUSSION

The dye-decolorizing peroxidase from *Amycolatopsis* 75iv2 (DyP2) was produced in the Gram-positive bacterium *S. lividans*. In previous works, *E. coli* was routinely used to produce bacterial peroxidases, which can be problematic because of a low yield of fully functional enzyme due to insufficient heme biosynthesis and/or incorporation (9–11, 14, 17, 19, 23–25, 27, 32, 34, 43–45). Only very recently, *E. coli* has been engineered to improve heme synthesis for the production of heme peroxidases (46). *S. lividans* displays a low extracellular protease activity, is phylogenetically more closely related to the gene source, *Amycolatopsis* 75iv2 (both belong to the class Actinomycetes), and its genome has a similarly high GC content (38). *S. lividans* TK 24 has been used recently to produce enzymes from other Actinomycetes such as laccases or pyranose oxidases (36, 37). The *DyP2*-encoding gene as annotated in the *Amycolatopsis* 75iv2 genome does not have a 5′-located sequence that appears to encode a signal peptide. Thus, DyP2 was initially produced intracellularly, which resulted in a protein with an *R*_*z*_ value of 1.4 ± 0.1 without any heme supplementation. The nutrients found in the phage medium, as the growth medium used, were sufficient to ensure heme synthesis for the production of the DyP2. The *R*_*z*_ value of the previously characterized DyP2 produced in *E. coli* was 1.6–1.8 (23), indicating that *S. lividans* can produce fully active peroxidase with proper heme incorporation using only the growth medium nutrients. Additionally, DyP2 intracellular production yielded 13 ± 2.8 mg of protein from a liter of culture which is comparable to the production yield of DyPs in *E. coli* (9, 43).

DyP2 is still the most active bacterial dye-decolorizing peroxidase characterized to date (23), and it has not been established whether *Amycolatopsis* 75iv2 harbors additional, comparably active, secretory peroxidases. The biological function of DyP2 is therefore still unclear. Since the high activity, comparable with fungal peroxidases, and the capability to oxidize Mn(II) (23) makes DyP2 an attractive enzyme for various biocatalytic applications, we attempted to also produce DyP2 in a secretory manner. There are *DyP*-encoding genes that contain signal peptide sequences (4, 5), and the N-terminal part of DtpA from *S. lividans* has been shown to be functional as a signal peptide (33). Sugawara and colleagues expressed two DyPs from *Streptomyces avermitilis*, both containing predicted putative N-terminal signal sequences. However, the enzymes were not secreted but accumulated intracellularly when produced in *S. lividans* (14, 44). We therefore used a construct from previous experiments on expression in *B. subtilis* (not shown) consisting of the DyP2 coding sequence fused with the sequence encoding the signal peptide of the *B. subtilis* dye-decolorizing peroxidase *Bs*DyP (11, 25, 43, 47). Indeed, the heterologous signal peptide was shown to be functional and resulted in secretory production of DyP2 in *S. lividans*.

This study focused on the previously unexplored DyP2 activity on plant *O*-glycosides. These compounds could be encountered by microorganisms during invasion as a defense response by plants (30, 48). Salicin, arbutin, fraxin, rutin, naringin, and gossypin present phenylic glycoside bonds. Salicin contains a non-phenolic aglycone and an ether-linked glycone (Fig. 2). No activity could be detected in reactions of DyP2 with salicin, confirming that DyP2 could not act on non-phenolic compounds without an additional mediator (23). Conversions of arbutin, fraxin, rutin, naringin, and gossypin with DyP2 resulted mainly in oligomerized products, which is in line with previously observed DyP reactions (8, 10). Compounds with the same *m*/*z* but various retention times were detected in all glycoside reactions, demonstrating the formation of isomers. Different fragmentation patterns and response to selected collision energies of arbutin oligomers further substantiated that those various isomeric compounds were formed upon C-C or C-O coupling. However, the type of bonds formed and the positions at which the oligomers are linked remained elusive.

Reactions on glycosides or on their aglycones have been performed with other oxidoreductases. Several studies have performed rutin polymerization using different laccases (49–52) and conversions of arbutin with peroxidases (53–56). Arbutin polymerization has also been performed using chemical catalysts (57, 58), resulting in enhanced anti-microbial properties. This study showed that arbutin can also be polymerized using dye-decolorizing peroxidases, which may have the advantage of controlling the degree of polymerization by modulating the concentration of H_2_O_2_, as well as milder operating conditions. Polyphenols are poorly soluble in water, but the solubility is improved with the glycone moiety (51).

**Fig 7:**
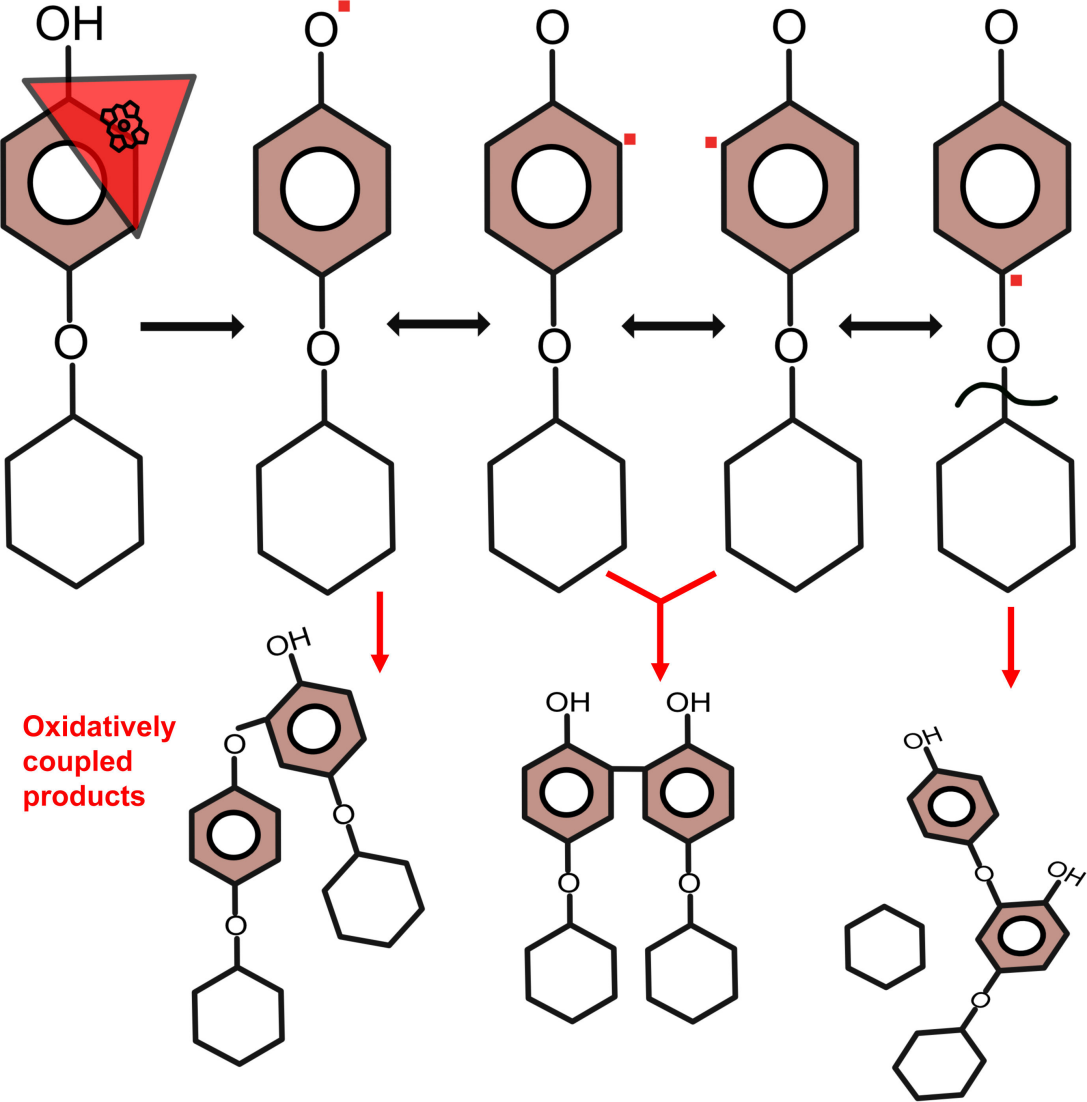
Proposed mechanism of radical mediated glycone release. DyP2 (red triangle) oxidizes the hydroxy group, but the radicals (red squares) can be distributed in the aromatic ring, resulting in many products of different isomers indicated by red arrows. When the radical is at the para position, then it could lead to destabilization of the ether linkage resulting in carbohydrate release.

Unexpectedly, mass spectrometry analysis of glycoside reaction products detected compounds with an *m*/*z* of 647 (arbutin), 552 (fraxin), and 912 (rutin), suggesting the loss of the glycone moiety. These samples were subjected to carbohydrate analysis using HPAEC. Free glycones were detected in the products of arbutin, fraxin, rutin, and gossypin conversions. Free glucose in samples of arbutin and fraxin reactions amounted to 23.5% and 3.0% of the total substrate, respectively. The differences in the amount of released glucose can be explained by chemical differences in the aglycone moiety. Fraxin has a structure composed of coumarin-like rings forming a conjugated system that DyP2 may favor for radical coupling. Arbutin has only one benzene ring, where a radical movement from the hydroxy group to the para position could destabilize the ether linkage and release the glycone (Fig. 7). The release of the glycone moiety is presumed to occur due to radicalization after favorable coupling since the primary products are dimers and trimers.

To the best of our knowledge, this research is the first to show carbohydrate release from *O*-glycosides as a result of enzymatic oxidation. DyP2 showed no activity with salicin, a non-phenolic aglycone linked with glucose via an ether bond, indicating that the carbohydrate release is a (non-enzymatic) consequence of the enzymatic oxidation of the phenolic moieties. Such a cleavage of ether-linked carbohydrates is not limited to dye-decolorizing peroxidases, as it was also observed in conversions of arbutin incubated with HRP. However, deglycosylation was dependent on the performed turnovers of the peroxidases since glucose release was detected after 15 min or 6,000 turnovers of H_2_O_2_ and increased when more turnovers were performed. In general, however, the glycone release from glycosides upon oxidative coupling using different oxidoreductases has not received a lot of attention or has been overlooked, as, e.g., Wang et al. showed that the sugar moieties remained in place after polymerization (59).

Since a phenyl glycosidic bond as present in many *O*-glycosides is one of the suggested bonds in LCCs, these small molecules could be used as model compounds to study the activity of enzymes like DyP on LCCs. Different lignocellulosic materials such as spruce, willow, wheat straw, and water-extractable lignin fractions were treated with DyP2. A color change and the formation of precipitate, in combination with high H_2_O_2_ turnovers, were observed in spruce and water-extractable lignin samples, indicating an enzyme activity presumably acting on unknown phenolic compounds. Moreover, pyrolysis-GC-MS confirmed the oxidative activity by detecting an increase in aldehyde, ketone, and diketone products. However, no significant changes were detected in the carbohydrate profile. The prevalence of LCC in natural settings is presumed to be low (31). Therefore, any changes caused by the enzyme could be difficult if not impossible to detect. It remains unclear at present if DyP2 truly acts on lignin-carbohydrate complexes. Nevertheless, applying dye-decolorizing peroxidases in biorefineries or food industry can lead to increased carbohydrate content and removal of polyphenols by forming insoluble oligomers based on model compounds.

## MATERIALS AND METHODS

### Chemicals

Rutin, naringin, arbutin, salicin, fraxin, gossypin, hydrogen peroxide, sodium acetate, acetic acid, imidazole, tris base, sodium chloride, magnesium sulfate, calcium chloride, glucose, tryptone, yeast extract, Lab Lemco powder, thiostrepton, ABTS, bovine serum albumin (BSA), Tween 20, Laemmli buffer were purchased from Sigma-Aldrich (Missouri, USA), if not stated otherwise.

### Protein production

*Amycolatopsis* 75iv2 dye-decoloizing peroxidase (WP_020421762.1) gene was codon optimized for *E. coli* and synthesized by General Biosystems (Durham, USA). The *E. coli* amplification plasmid was constructed using Gibson Assembly (New England Biolabs, Massachusetts, USA) according to the manufacturer’s protocol. Primers 5′-TCGATCGAAGGAGAGCTGCAATGCCGGTTGATCTGAGCAC-3′ and 5′-TCCTCTAGAGTCGACCTGCATTAGTGGTGATGGTGATGATGC-3′ were used to generate overhangs to the *DyP2* coding region with PCR. *E. coli* plasmid pUC-P_vsi_ was linearized with *Pst*I, incubated with the insert containing the overhangs for 30 min at 50°C, and transformed into chemically competent *E. coli* JM109. pUC-P_vsi_-*DyP2* and the expression plasmid pIJ486 were both digested with *Hind*III and *Xba*I, ligated with T4 ligase, and transformed into *S. lividans* TK24 protoplasts as described in reference (60). Positive clones were grown in a phage medium (0.5 g/L MgSO_4_ 7H_2_O; 0.74 g/L CaCl_2_ 2H_2_O; 10 g/L glucose; 5 g/L tryptone; 5 g/L yeast extract; and 5 g/L Lab Lemco powder, pH 7.2) containing 10 µg/mL thiostrepton, and constructs were verified by Sanger sequencing (Microsynth AG, Balgach, Switzerland). *S. lividans* spores were prepared as described in reference (60) and were used for intracellular enzyme production in 1 L baffled flasks. Approximately 10,000 CFU/mL spores were inoculated into a 200 mL phage medium containing 10 µg/mL thiostrepton, cultured at 28°C at 140 rpm for 3–4 days and harvested by centrifugation at 9,500 rpm for 30 min. The cell pellet was resuspended in buffer A (50 mM Tris 300 mM NaCl pH 7.4), sonicated for 1 min at 80 V and 70% cycles using a Bandelin Sonopuls HD 60 (Bandelin Electronic GmbH, Berlin, Germany), centrifuged at 20,000 rpm for 30 min and filtered through 0.22 µm. Cell lysate was loaded onto HisTrap (5 mL) column (GE Healthcare, Chicago, USA) at a rate of 2 mL/min speed, washed with buffer A until UV (280 nm) stabilized and eluted with a linear gradient of 0–100% with buffer B (50 mM Tris 300 mM NaCl 500 mM imidazole pH 7.4). Enzyme fractions were pooled and dialyzed against 20 mM Tris 150 mM NaCl pH 7.4 using 14,000 Da molecular weight cut off cellulose membrane (Sigma-Aldrich) and stored at −80°C.

Extracellular DyP2 was produced using the signal sequence from *B. subtilis* DyP (WP_003243445). The signal sequence was amplified from *B. subtilis* genomic DNA using 5′-TCGATCGAAGGAGAGCTGCAATGAGCGATGAACAGAAAAAGCC-3′ and 5′-AGCCGCAGTCTGAACAAGCG-3′ primers and cloned with Gibson Assembly (New England Biolabs) as described above. SP-DyP2 was recloned into pIJ486, transformed into *S. lividans* protoplasts, and protein was produced using *S. lividans* cell culture. Preculture (20 mL phage medium with 10 µg/mL thiostrepton) was cultured for three to four days at 28°C 140 rpm, two mL was inoculated into fresh 200 mL phage medium with 10 µg/mL thiostrepton in one-liter baffled flasks. Protein production was conducted for two to three days at 28°C 140 rpm, the supernatant was harvested by centrifugation, filtered and pH was adjusted with 10 x concentrated buffer A before loading onto the purification column. Purification was conducted as described above.

### SDS-PAGE and Western blot

Protein was mixed with 2 x Laemmli buffer, denatured at 95°C for 10 min, loaded in Mini-PROTEAN TGX Stain-Free Precast Gels 4–20% (Bio-Rad, Hercules, USA), and ran at 120 V for 60 min. Precision Plus Protein Western C (Bio-Rad) was used as a molecular weight standard. The gel was transferred to Bio-Rad Western blot membrane using dry blotting in a Trans-Blot Turbo (Bio-Rad) at 1.3 A and 25 V for 7 min. Blotted membrane was immediately incubated with 5% BSA for one hour at room temperature. Next, the membrane was incubated with primary antibody (anti-penta-his tag) (Qiagen, Hilden, Germany) overnight at 4°C 100 rpm, washed with tris buffered saline with Tween 20 (TBST) three times, incubated with secondary antibodies (Polyclonal rabbit-anti-mouse immunoglobulin/HRP) (Agilent, Santa Clara, USA) for one hour, washed with TBST three times, incubated with HRP substrate Clarity Western ECL (Bio-Rad) for 5 min, and visualized with ChemiDoc XRS+ (Bio-Rad).

### Activity assay

Preculture (20 mL phage medium and 10 µg/mL thiostrepton) was started from a cryoculture and was cultured for 3 to 4 days at 28°C 140 rpm. One mycelial cluster wacos inoculated into fresh 50 mL phage medium with 10 µg/mL thiostrepton in 300 mL baffled flasks and incubated at 28°C 140 rpm. One mL of the culture was harvested every day for four days. The samples were centrifuged for 15 min at 4°C 10,000 rpm. The pellet was washed twice with 0.9% NaCl solution, centrifuged for 7 min at 4°C 15,000 rpm and resuspended in the initial sample volume of 1 mL of 20 mM Tris-HCl (pH 7.4) buffer. The cells were lysed using Sonopuls HD60 (Bandelin Electronic GmbH) for 40–50 s at 80 V and 50% cycles and centrifuged for 7 min at 4°C at 15,000 rpm. The supernatant and cell extract were used in the activity assays.

DyP activity in the culture medium was measured using ABTS as the substrate. The oxidation of ABTS was detected by measuring the absorbance at 420 nm (*ε*_420_ = 36,000 M^−1^ cm^−1^). The reaction mixture (300 µL) contained 30 µL of 25 mM ABTS (final concentration 2.5 mM), 60 µL of enzyme (supernatant or cell extract), 30 µL of 5 mM (final concentration 0.5 mM), and 180 µL of 50 mM sodium acetate (pH 4.5) buffer. ABTS and H_2_O_2_ solutions were prepared fresh prior to each measurement. The reactions were measured using plate reader EnSpire multimode (PerkinElmer, Shelton, USA). The supernatant and cell extract from the strain containing the empty plasmid and growth medium were used as negative controls.

### Enzyme incubations with glycosides

Reactions were conducted in 2 mL of Eppendorf tubes. Soluble glycosides (arbutin and fraxin 2.6 or 0.26 mM) and insoluble glycosides (rutin, naringin, and gossypin 1 g/L) were incubated with 0.2 µM of DyP2, and H_2_O_2_ of 200 µM was added fresh after 3 min for 60 min. The 3 min time interval was chosen based on preliminary measurements with an H_2_O_2_ sensor (61). Sample points were taken at 3, 15, 30, 45, and 60 min. Samples were analyzed with UHPLC-MS, IQ-X, MALDI-TOF-MS, and HPAEC as described below. The H_2_O_2_ turnovers were calculated: *n* = *c*(H_2_O_2_) / *c*(DyP2), where *c* is the concentration in micromolar.

### Biomass treatment and enzyme incubation

Wheat straw (Vienna, Austria) was milled to obtain a particle size of <125 µm. Spruce (Wageningen, The Netherlands) and willow (Wageningen, The Netherlands) samples were obtained from previously published work (42). Briefly, wood samples had been coarsely milled, extracted with acetone and water, and subsequently planetary ball milled. Two purified water-soluble lignin fractions (S2 and S4) from wheat straw, before and after planetary ball milling of the biomass, respectively, were obtained from a previous work (62). Plant material (100 g/L) or water-extractable lignin fractions (5 g/L) were resuspended in sodium acetate buffer (pH 4.5); 2 µM enzyme was added, and the reaction was started with 200 µM H_2_O_2_. The reaction was continued by adding fresh H_2_O_2_ after every 3 min for 60 min. The substrate with H_2_O_2_ and inactivated DyP2 was used as a control. Controls and enzymatically treated samples were analyzed by pyrolysis-gas chromatography-mass spectrometry.

### Pyrolysis-GC-MS

Control and DyP-treated water-soluble lignin residues were freeze-dried and qualitatively analyzed in single replicates by pyrolysis-GC-MS as published (42), but importantly without internal standard being added. Relative abundances of lignin-derived pyrolysis products were calculated as reported, but logically without any internal standard correction.

### Reversed-phase liquid chromatography ion trap mass spectrometry (RP-UHPLC-PDA-IT-MS)

Compounds were separated on an Vanquish UHPLC reversed-phase (RP) system (Thermo Scientific, Massachusetts, USA) equipped with a pump, autosampler, and photodiode array (PDA) detector. The undiluted samples (3 µL) were injected onto an Acquity UHPLC BEH C18 column (150.0 mm × 2.1 mm, particle size 1.7 µm) (Waters) equipped with a VanGuard guard column (5.0 mm × 2.1 mm, particle size 1.7 µm) of the same material. The flow rate was 300 µL/min at 45°C. Premixes containing water (A) and acetonitrile (B) both acidified with 0.1% formic acid (Biosolve, Valkenswaard, The Netherlands) were used as eluents. The following elution profile was used: 0–2.6 min, 5% B (isocratic); 2.6–45.1 min, from 5% to 39% B (linear gradient); 45.1–46.1 min, from 39% to 100% B (linear gradient); 46.1–51.1 min, 100% B (isocratic); 51.1–52.1 min, from 100% to 5% B (linear gradient); and 52.1–58.1 min, 5% B (isocratic). The PDA detector was set to record spectra between 200 and 600 nm.

Mass spectrometric data were obtained using Velos Pro linear ion trap mass spectrometer (Thermo Scientific) equipped with a heated ESI probe coupled with the Vanquish UHPLC system. Nitrogen was used as sheath gas and auxiliary gas. Data were collected in negative ionization mode over the *m*/*z* range of 100–2,000. Data-dependent MS^2^ analysis was performed using CID with a normalized collision energy (NCE) of 35%. The ion transfer tube temperature was 300°C; the source heater temperature was 250°C; and the source voltage was 2.5 kV. The data were processed using Freestyle (Thermo Scientific).

Arbutin MS^2,3,4^ was fragmented following a mass list. Masses *m*/*z* 377, 541, 557, 587, 613, 647, 693, 811, 917, and 1,081 from the full MS scan were selected for MS^2^, and MS^3,4^ fragmentation was obtained from the most abundant fragment of MS^n−1^.

### Accurate mass determination by RP-UHPLC-VWD-IT-FT-MS analysis

Compounds from the arbutin 15-min sample point were separated on an a Vanquish UHPLC reversed-phase system (Thermo Scientific) equipped with a pump, autosampler, and variable wavelength detector (VWD). The sample was diluted two times, and 1 µL was injected onto an Acquity UHPLC BEH C18 column (150.0 mm × 2.1 mm, particle size 1.7 µm) (Waters) equipped with a VanGuard guard column (5.0 mm × 2.1 mm, particle size 1.7 µm) of the same material. The flow rate was 300 µL/min at 45°C. Premixes (Biosolve) containing water (A) and acetonitrile (B) both acidified with 0.1% formic acid were used as eluents. The following elution profile was used: 0–1.5 min, 1% B (isocratic); 1.5–30.8 min, from 1% to 18% B (linear gradient); 30.8–32.2 min, from 18% to 100% B (linear gradient); 32.2–39.5 min, 100% B (isocratic); 39.5–41.0 min from 100% to 1% B (linear gradient); and 41.0–48.5 min, 1% B (isocratic). The VWD detector was set to 280 nm.

Accurate mass data were obtained using Orbitrap IQ-X Tribrid mass spectrometer (Thermo Scientific) equipped with a heated ESI (HESI) probe and coupled to the Vanquish system. Nitrogen was used as sheath gas and auxiliary gas. HESI default settings were used based on the UHPLC flow. The ion transfer tube temperature was 325°C; the source heater temperature was 300°C; and the source voltage was 2.5 kV. Before analysis, the IQ-X was calibrated with the Auto-Ready system using the Pierce FlexMix Calibration Solution for Auto Ready Mass Spectrometers (Thermo Scientific). Fluoranthene was used as the internal EASY-IC calibrant for full MS spectra. Full MS data were collected in negative ionization mode over the *m*/*z* range of 500–1,000 at 60,000 full width at half maximum (FWHM) resolution.

MS^2^ analysis was performed using HCD 15 NCE, 25 NCE, and 35 NCE energy using a precursor ion inclusion mass list: 541.156, 587.161, 647.161, 693.167, 811.230, 857.235, and 917.236. Mass tolerance for both high and low were set at 5 ppm. The top three data-dependent MS^3^ spectra were collected in CID using 35% energy. The mass resolutions for MS^2^ spectra and MS^3^ spectra were set at 30,000 FWHM. All MS^n^ spectra were recorded in profile mode. The data were processed using Freestyle version 1.8 (Thermo Scientific). Theoretical masses were calculated using https://www.sisweb.com/referenc/tools/exactmass.htm.

### MALDI-TOF MS

Fraxin insoluble fraction was solubilized in 80 µL of MeOH and 10 µL of DMSO. Arbutin products were completely water soluble, thus analyzed as they were. Sample spots were prepared by mixing 1 µL 2,5-dihydroxy-benzoic acid matrix substance (12 g/L in 50% acetonitrile) with a 1 µL sample on a MTP384 ground steel target plate (Bruker Daltonics, Massachusetts, USA). Drying was accelerated using a hairdryer. A total of 1,500 spectra in the *m*/*z* range of 300–3,000 were obtained in positive mode using an Ultraflextreme workstation (Bruker Daltonics), equipped with a Smartbeam II laser of 355 nm. A laser intensity of 50%–75% was used with ion source voltages of 20.0 and 17.86 kV, reflector voltages of 20 and 11 kV, and lens voltage of 7.59 kV. The results were analyzed using FlexAnalysis (Bruker Daltonics).

### HPAEC

The analysis was performed on an ICS-5000 system (Dionex, Thermo Scientific) equipped with CarboPac PA-1 column (2 mm ID × 250 mm, Dionex) in combination with a CarboPac PA guard column (2 mm ID × 50 mm, Dionex). The mobile phases were 0.1 M NaOH (A) and 1 M NaOAc in 0.1 M NaOH (B). The column temperature was set to 20°C, and the elution profile was set at 0–50 min, from 0% to 50% B (linear gradient); 50–60 min, 100% B (isocratic); and 60–70 min, from 100% to 0% B (isocratic). The peak areas were manually integrated for quantification. The starting substrate concentration (2.6 or 0.26 mM) was set as 100%, and glucose release was calculated as percentage of the substrate concentration. Incubations were performed in duplicate.

## SUPPLEMENTAL MATERIAL

Supplemental material aem.00205-24-s0001.docx Figures S1 to S18; Tables S1 to S7.

ASM does not own the copyrights to Supplemental Material that may be linked to, or accessed through, an article. The authors have granted ASM a non-exclusive, world-wide license to publish the Supplemental Material files. Please contact the corresponding author directly for reuse.
