# Supplementary material for: Characterization of Amycolatopsis 75iv2 dye-decolorizing peroxidase on O-glycosides
Source: Appl Environ Microbiol. 2024 Apr 16;90(5):e00205-24. doi: 10.1128/aem.00205-24 (PMC11107159; doi:10.1128/aem.00205-24)
Supplement: Supplemental material — Figures S1 to S18; Tables S1 to S7. [file aem.00205-24-s0001.docx]

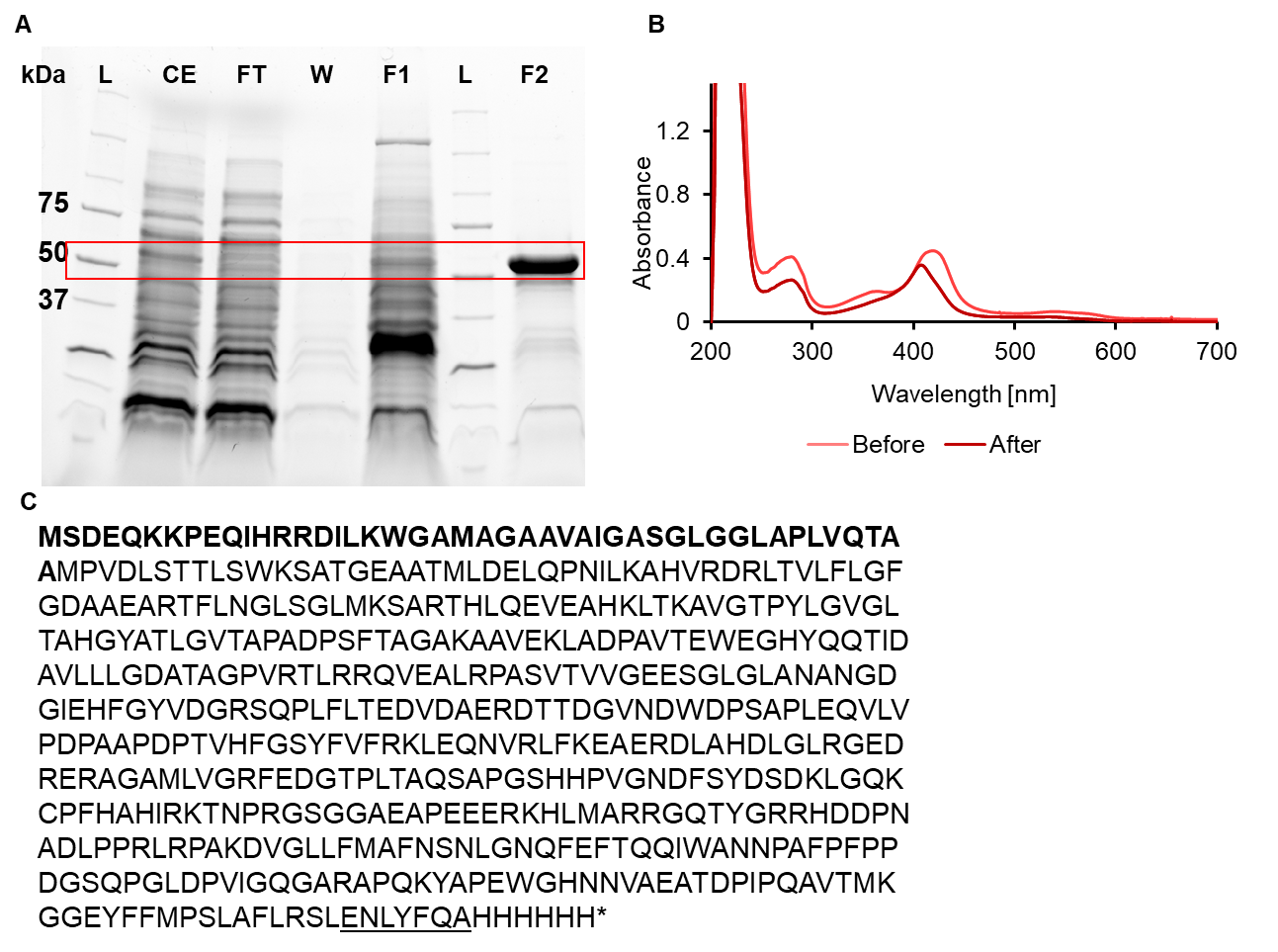


**Supplementary Figure 1: Intracellular production of DyP2 in Streptomyces lividans.** **A)** SDS-PAGE analysis of DyP2-TEV-His_6_. L-ladder, CE-crude extract, FT- flow through during column loading, W- column wash, F1- purification fraction 1, F2- purification fraction 2. DyP2 expected size based on theoretical calculated molecular weight is 52 kDa. F2 was loaded at high concentration to estimate the purity of the protein. **B)** UV-Vis spectra of DyP2 before and after dialysis. The Soret band was at maximum 429 nm before dialysis suggesting a haem-bound imidazole, but after dialysis the Soret band shifted to 410 nm indicating removal of imidazole. **C)** Amino acid sequence of the DyP2 construct with Bacillus subtilis A- type DyP signal peptide (marked in bold). Tobacco Etch Virus protease cleavage site is underlined, and C-terminus contains His_6_ for downstream purification using affinity chromatography.


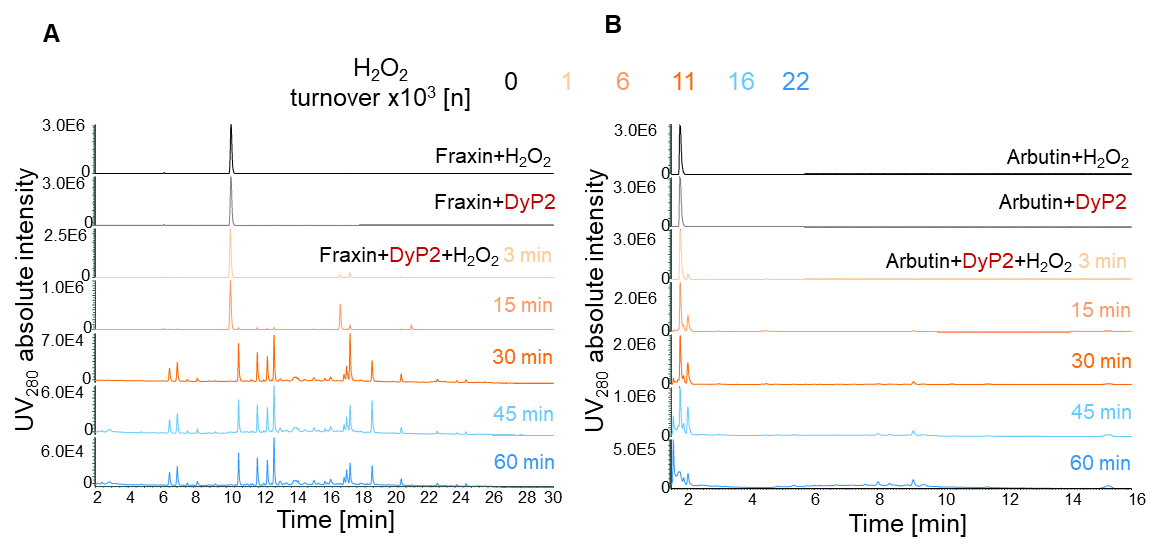


**Supplementary Figure 2: Time course UV (280 nm) chromatograms. A)** DyP2 incubation with fraxin and **B)** DyP2 incubation with arbutin. The substrates with final concentration of 2.6 mM were solubilised in a sodium acetate buffer at pH 4.5 containing 0.2 µM enzyme, and the reaction was initiated with the addition of 200 µM H_2_O_2_. The reaction was continued by adding fresh H_2_O_2_ after every three minutes for 60 minutes. H_2_O_2_ turnovers per time point is colour coded above the figures. Samples were taken after 3, 15, 30, 45 and 60 minutes. Substrates containing only H_2_O_2_ or DyP2 were used as controls. The incubations were conducted in duplicates but only one of the identical replicates is shown. Tentatively annotated products of fraxin and arbutin incubations are listed in Supplementary Table 1 and 2.


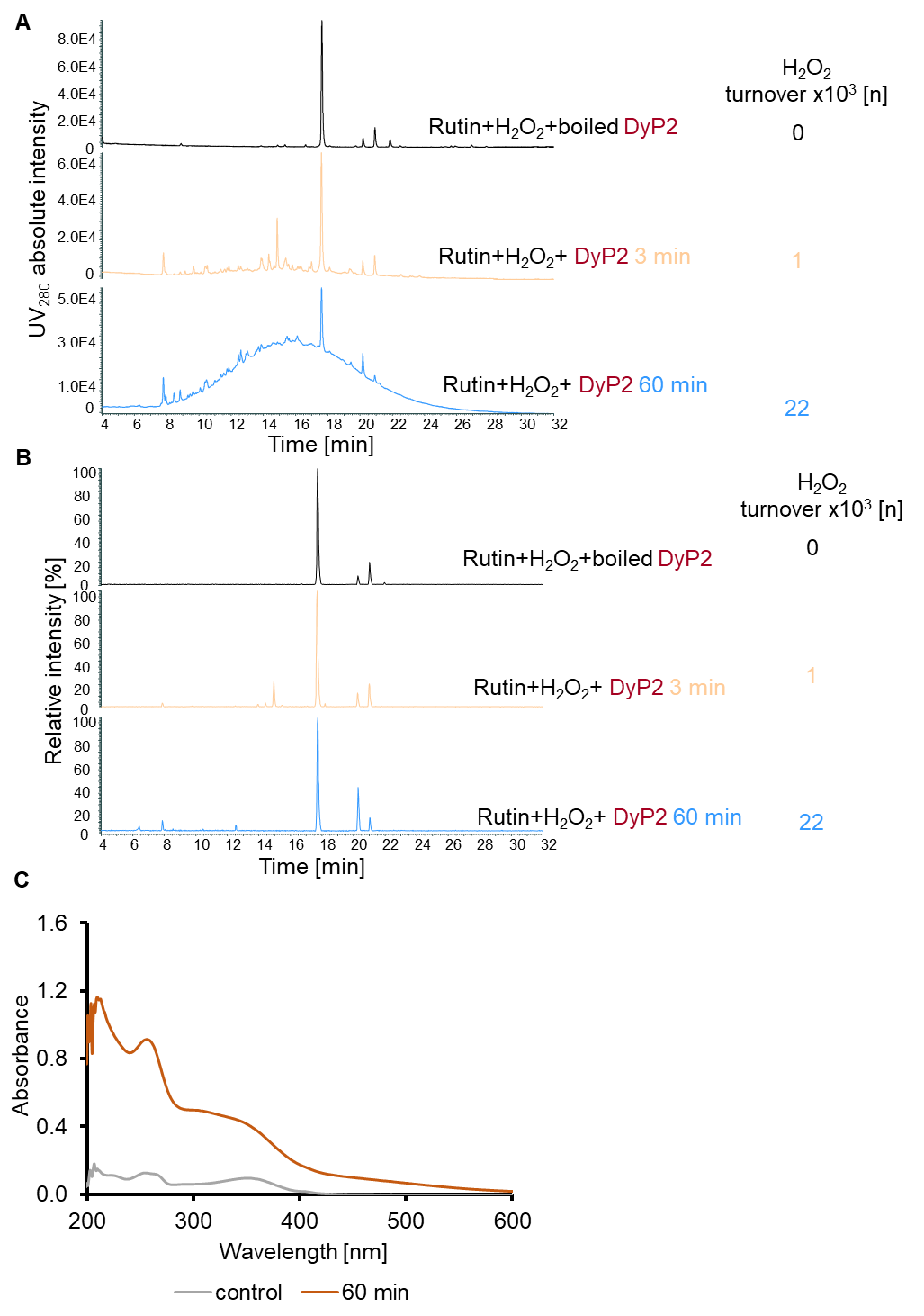


**Supplementary Figure 3: DyP2 incubations with partially soluble rutin. A)** UV (280 nm) chromatograms at 3 and 60 minutes. Rutin 1 g L^-1^ was resuspended in 50 mM sodium acetate buffer pH 4.5, 0.2 µM enzyme was added, and the reaction was initiated with 200 µM H_2_O_2._ After every three minutes fresh 200 µM H_2_O_2_ was added in the reaction for 60 minutes. Rutin with H_2_O_2_ and inactivated DyP2 was used as a control. The incubations were performed in duplicates but only one of the identical replicates is shown. After three minutes, many new peaks were detected indicating heterogeneous reaction products. After 60 minutes, no clear separation of products was observed resulting in hill-like chromatogram. **B)** UHPLC-MS negative mode chromatograms at 3 and 60 minutes. Few UV detected compounds were ionized and detected in the mass analyser. **C)** UV-Vis spectra of rutin before and after incubation with DyP2.


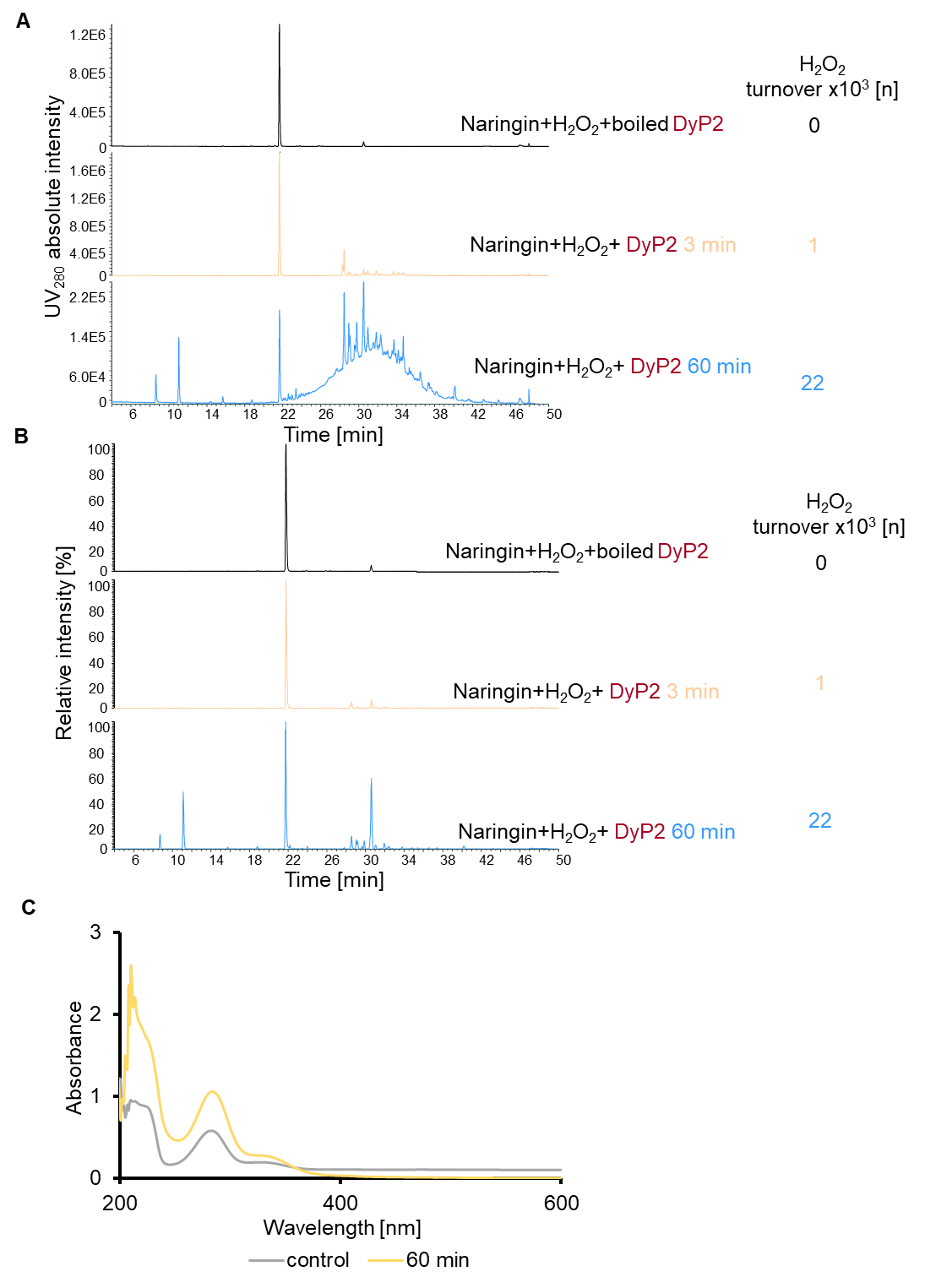


**Supplementary Figure 4:** **DyP2 incubations with partially soluble naringin. A)** UV (280 nm) chromatograms at 3 and 60 minutes. Naringin 1 g L^-1^ was resuspended in 50 mM sodium acetate buffer pH 4.5, 0.2 µM enzyme was added, and the reaction was initiated with 200 µM H_2_O_2_. After every three minutes fresh 200 µM H_2_O_2_ was added in the reaction for 60 minutes. Naringin with H_2_O_2_ and inactivated DyP2 was used as a control. The incubations were performed in duplicates but only one of the identical replicates is shown. After three minutes, many new peaks were detected indicating heterogeneous reaction products. After 60 minutes, no clear separation of products was observed resulting in hill-like chromatogram. **B)** UHPLC-MS negative mode chromatograms at 3 and 60 minutes. Few UV detected compounds were ionized and detected in the mass analyser. **C)** UV-Vis spectra of naringin before and after incubation with DyP2.


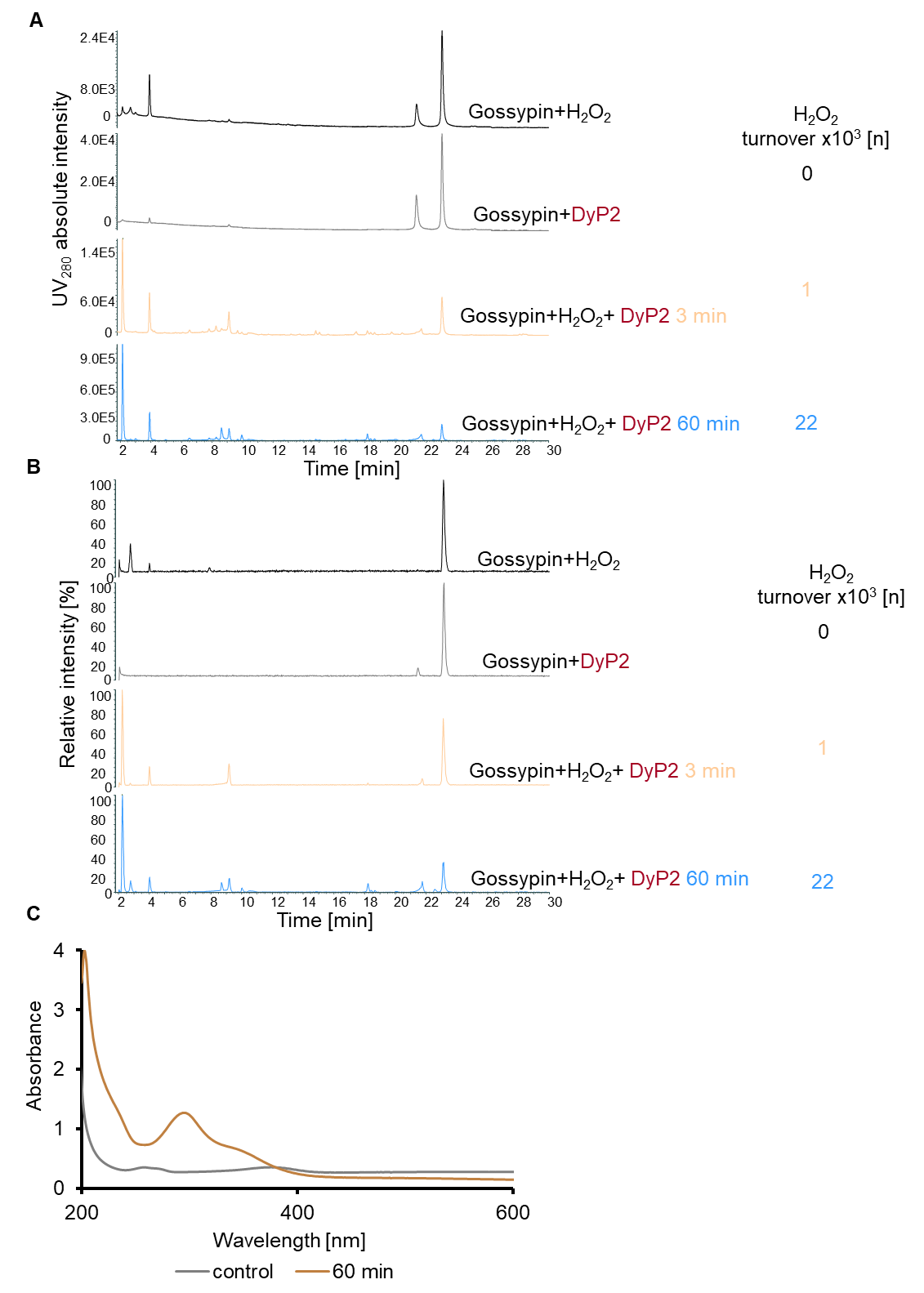


**Supplementary Figure 5: DyP2 incubations with partially soluble gossypin. A)** UV (280 nm) chromatograms at 3 and 60 minutes. Gossypin 1 g L^-1^ was resuspended in 50 mM sodium acetate buffer pH 4.5, 0.2 µM enzyme was added, and the reaction was initiated with 200 µM H_2_O_2_. After every three minutes fresh 200 µM H_2_O_2_ was added in the reaction for 60 minutes. Gossypin with H_2_O_2_ or DyP2 only were used as controls. The incubations were performed in duplicates but only one of the identical replicates is shown. After three minutes, new peaks were detected. After 60 minutes more peaks were detected indicating heterogeneous reaction products. **B)** UHPLC-MS negative mode chromatograms at 3 and 60 minutes. **C)** UV-Vis spectra of gossypin before and after incubation with DyP2.


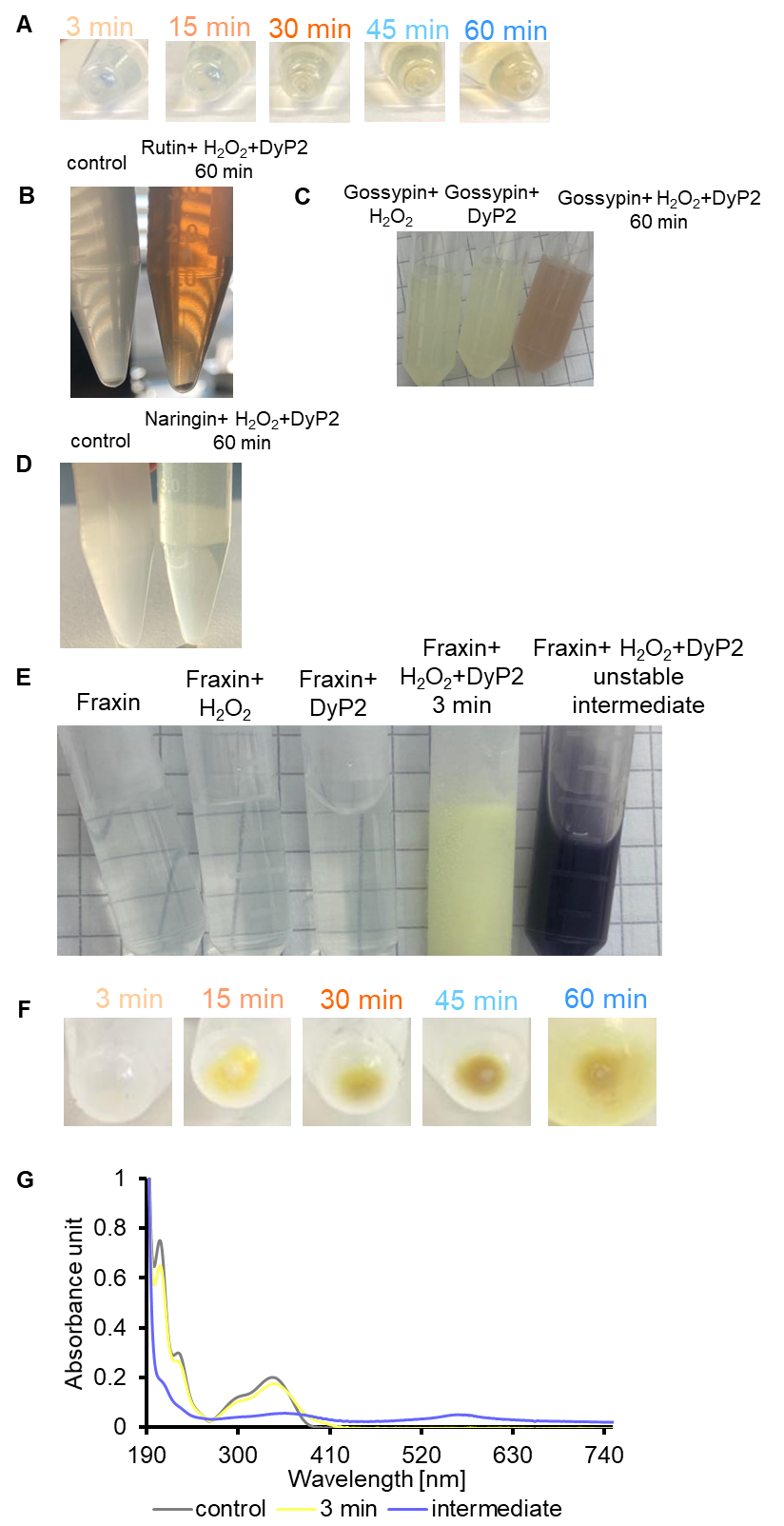


**Supplementary Figure 6:** **Visualisation of O-glycoside incubations**. **A)** Time course of colour formation in arbutin incubations. After 60 min all products were still completely water soluble. **B)** Colour change in rutin incubations with and without DyP2. **C)** Colour change in gossypin incubations with and without DyP2. **D)** Colour change in naringin incubations with and without DyP2. **E)** Fully soluble fraxin was colourless, but the colour changed to yellow after initiation with 0.2 mM H_2_O_2_. A blue-violet unstable intermediate was formed after 30 minutes. The intermediate persisted for one hour and then changed back to yellow-green colour. **F)** Time course of the formation of the fraxin insoluble fraction. No precipitate was formed after 3 minutes. The pellets were solubilised in MeOH/DMSO mixture and analysed with MALDI-TOF-MS. **G)** UV-Vis spectra of fraxin control, three min time point and unstable blue intermediate after incubation with DyP2.


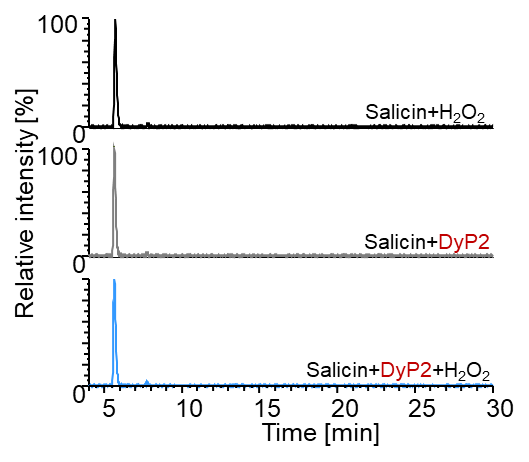


**Supplementary Figure 7: UHPLC-MS negative mode chromatogram of salicin incubation**. Salicin 1 g L^-1^ was resuspended in 50 mM sodium acetate buffer pH 4.5, 0.2 µM enzyme was added, and the reaction was initiated with 200 µM H_2_O_2_. After every three minutes fresh 200 µM H_2_O_2_ was added in the reaction for 60 minutes. Salicin with H_2_O_2_ and inactivated DyP2 was used as a control. The incubations were performed in duplicates but only one of the identical replicates is shown. As expected, no activity was observed with non-phenolic glycoside when incubated with DyP2.

**Supplementary Table 1:** **Compounds detected with UHPLC-MS after incubation of fraxin with DyP2**. R.A= relative abundance, ND= not determined.

| **RT** | **Tent. Annot.** | **UV_max_ [nm]** | **Ion** | **m/z** | **R.A** | **MS^2^** | **R.A** | **MS^2^** | **R.A** |
| --- | --- | --- | --- | --- | --- | --- | --- | --- | --- |
| 3 | Unknown | 204 | [M-H]^-^ | 741 | 100 | 343 | 100 | 713 | 43 |
| 6.33 | Unknown | 336 | [M-H]^-^ | 339 | 100 | 177 | 100 |  |  |
| 6.63 | Dimer | 228 | [M-H]^-^ | 737 | 100 | 557 | 100 | 531 | 38 |
| 7.13 | Dimer | 228 | [M-H]^-^ | 737 | 100 | 557 | 100 | 559 | 56 |
| 8.37 | Unknown | 204 | [M-H]^-^ | 531 | 100 | 207 | 100 | 192 | 43 |
| 10.42 | Fraxin | 332 | [M-H]^-^ | 369 | 100 | 207 | 100 | 192 | 13 |
| 10.87 | Oligomer-Glucose | 232 | [M-H]^2-^ | 552 | 100 | 463 | 100 | 381 | 29 |
| 10.87 | Oligomer-Glucose | 232 | [M-H]^-^ | 1105 | 85 | 925 | 100 | 899 | 53 |
| 11.25 | Unknown | 228 | [M-H]^-^ | 531 | 100 | ND |  |  |  |
| 11.99 | Oligomer-Glucose | 228 | [M-H]^2-^ | 552 | 100 | 463 | 100 | 471 | 36 |
| 11.99 | Oligomer-Glucose | 228 | [M-H]^-^ | 1105 | 50 | 925 | 100 | 899 | 61 |
| 12.63 | Oligomer-Glucose | 228 | [M-H]^-^ | 1105 | 100 | 925 | 100 | 763 | 20 |
| 13.05 | Oligomer-Glucose | 228 | [M-H]^-^ | 1105 | 100 | 925 | 100 | 763 | 34 |
| 16.52 | Unknown | 228 | [M-H]^2-^ | 1100 | 100 | 737 | 100 | 899 | 15 |
| 17.11 | Dimer | 232 | [M-H]^-^ | 737 | 100 | 575 | 100 | 413 | 28 |
| 17.72 | Dimer | 380 | [M-H]^-^ | 737 | 100 | 575 | 100 | 413 | 8 |
| 19.06 | Unknown | 228 | [M-H]- | 709 | 100 | 547 | 100 |  |  |
| 20.81 | Oligomer-Glucose | 232 | [M-H]^2-^ | 552 | 100 | 471 | 100 | 390 | 10 |
| 20.81 | Oligomer-Glucose | 232 | [M-H]^-^ | 1105 | 14 | 943 | 100 | 573 | 47 |
| 21.44 | Oligomer-Glucose | 232 | [M-H]^-^ | 1105 | 100 | 943 | 100 | 781 | 81 |
| 23.02 | Unknown | 228 | [M-H]^2-^ | 736 | 100 | 655 | 100 | 574 | 50 |

**Supplementary Table 2: Compounds detected with UHPLC-MS after incubation of arbutin with DyP2**. R.A= relative abundance, ND= not determined. The most abundant MS^n^ fragments are shown.

| **RT** | **Tent. Annot.** | **UV_max_ [nm]** | **Ion** | **m/z** | **R.A** | **MS^2^** | **MS^3^** | **MS^4^** |
| --- | --- | --- | --- | --- | --- | --- | --- | --- |
| 1.87 | Arbutin | 280 | [M+HCOO^-^]^-^ | 317 | 100 | 271 |  |  |
| 1.97 | Trimer | 232 | [M-H]^-^ | 811 | 100 | 649 | 486 | 324 |
| 2.12 | Dimer | 232 | [M-H]^-^ | 541 | 100 | 379 | 217 | 199 |
| 2.12 | Unknown | 232 | [M-H]^-^ | 1083 | 26 | ND |  |  |
| 3.07 | Trimer | 208 | [M-H]^-^ | 811 | 100 | 649 | 487 | 325 |
| 3.07 | Unknown | 208 | [M-H]^-^ | 1081 | 32 | 919 | 757 | 595 |
| 4.45 | Trimer | 228 | [M-H]^-^ | 811 | 100 | 649 | 487 | 393 |
| 4.59 | Dimer | 228 | [M+HCOO^-^]^-^ | 587 | 100 | 541 | 379 | 217 |
| 4.59 | Dimer | 228 | [M-H]^-^ | 541 | 63 | 379 | 217 | 123 |
| 5.47 | Unknown | 208 | [M-H]^-^ | 377 | 100 | 215 | 171 | 143 |
| 5.47 | Unknown | 228 | [M-H]^-^ | 1125 | 64 | ND |  |  |
| 8.11 | Trimer-glucose | 228 | [M-H]^-^ | 647 | 100 | 485 | 323 | 305 |
| 8.47 | Unknown | 228 | [M-H]^-^ | 917 | 100 | 755 | 593 | 430 |
| 8.94 | Trimer | 228 | [M+HCOO^-^]^-^ | 857 | 100 | ND |  |  |
| 8.94 | Trimer | 228 | [M-H]^-^ | 811 | 85 | 487 | 325 | 215 |
| 9.21 | Trimer-glucose | 228 | [M-H]^-^ | 647 | 100 | 485 | 323 | 305 |
| 9.49 | Unknown | 228 | [M-H]^-^ | 917 | 100 | 755 | 593 | 431 |
| 9.49 | Unknown | 228 | [M+HCOO^-^]^-^ | 963 | 45 | ND |  |  |
| 11.56 | Unknown | 228 | [M-H]^-^ | 917 | 100 | 755 | 593 | 431 |
| 15.36 | Trimer-glucose | 228 | [M+HCOO^-^]^-^ | 693 | 100 | 647 | 485 | 323 |
| 15.36 | Trimer-glucose | 228 | [M-H]^-^ | 647 | 40 | 485 | 323 | 305 |

**Supplementary Table 3:** **Compounds detected with UHPLC-MS after incubation of rutin with DyP2**. R.A= relative abundance, ND= not determined.

| **RT** | **Tent. Annot.** | **UV_max_** | **m/z** | **Ion** | **R.A** | **MS^2^** | **R.A** | **MS^2^** | **R.A** |
| --- | --- | --- | --- | --- | --- | --- | --- | --- | --- |
| 6.38 | Unknown | 228 | 517 | [M-H]^-^ | 100 | 471 | 100 | 489 | 47 |
| 7.89 | Trimer-Rutinose | 228 | 912 | [M-H]^2-^ | 100 | 758 | 100 | 604 | 22 |
| 12.54 | Unknown | 228 | 562 | [M-H]^2-^ | 100 | 399 | 100 | 386 | 33 |
| 13.96 | Dimer | 228 | 608 | [M-2H]^2-^ | 100 | 454 | 100 |  |  |
| 13.96 | Dimer | 228 | 1217 | [M-H]^-^ | 21 | ND |  |  |  |
| 14.43 | Dimer | 228 | 608 | [M-2H]^2-^ | 100 | 454 | 100 | 300 | 15 |
| 14.43 | Dimer | 228 | 1217 | [M-H]^-^ | 20 | ND |  |  |  |
| 14.97 | Dimer | 228 | 608 | [M-2H]^2-^ | 100 | 454 | 100 | 300 | 16 |
| 14.97 | Dimer | 228 | 1217 | [M-H]^-^ | 15 | ND |  |  |  |
| 17.73 | Rutin | 228 | 609 | [M-H]^-^ | 100 | 301 | 100 |  |  |
| 18.22 | Unknown | 228 | 667 | [M-H]^-^ | 100 | 299 | 100 | 271 | 26 |
| 20.28 | Unknown | 228 | 593 | [M-H]^-^ | 100 | 285 | 100 |  |  |
| 21.03 | Unknown | 228 | 623 | [M-H]^-^ | 100 | 315 | 100 | 300 | 17 |

**Supplementary Table 4:** **Compounds detected with UHPLC-MS after incubation of naringin with DyP2**. R.A= relative abundance, ND= not determined.

| **RT** | **Tent. Annot.** | **UV_max_** | **m/z** | **Ion** | **R.A** | **MS^2^** | **R.A** | **MS^2^** | **R.A** |
| --- | --- | --- | --- | --- | --- | --- | --- | --- | --- |
| 8.75 | Unknown | 228 | 503 | [M-H]^-^ | 100 | 485 | 100 | 475 | 16 |
| 11.17 | Unknown | 256 | 531 | [M-H]^-^ | 100 | 485 | 100 |  |  |
| 11.17 | Unknown | 256 | 485 | [M-H]^-^ | 40 | 177 | 100 | 365 | 22 |
| 11.17 | Unknown | 256 | 1017 | [M-H]^-^ | 19 | 971 | 100 | 485 | 54 |
| 15.79 | Unknown | 228 | 785 | [M-H]^-^ | 100 | 577 | 100 |  |  |
| 18.87 | Unknown | 228 | 595 | [M-H]^-^ | 100 | 459 | 100 |  |  |
| 21.77 | Naringin | 284 | 579 | [M-H]^-^ | 100 | 459 | 100 | 271 | 30 |
| 22.23 | Unknown | 228 | 577 | [M-H]^-^ | 100 | 269 | 100 |  |  |
| 24.04 | Unknown | 228 | 623 | [M-H]^-^ | 100 | 459 | 100 | 315 | 97 |
| 28.56 | Dimer | 232 | 1157 | [M-H]^-^ | 100 | 697 | 100 | 723 | 25 |
| 28.56 | Dimer | 232 | 578 | [M-2H]^2-^ | 65 | 424 | 100 |  |  |
| 28.56 | Dimer | 232 | 601 | [M-2H+Na^+^]^2-^ | 50 | 578 | 100 |  |  |
| 29.04 | Dimer | 232 | 1203 | [M+HCOO^-^]^-^ | 100 | 1157 | 100 | 1182 | 94 |
| 29.04 | Dimer | 232 | 1157 | [M-H]^-^ | 37 | 671 | 100 |  |  |
| 29.11 | Dimer | 232 | 1203 | [M+HCOO^-^]^-^ | 100 | 1157 | 100 | 671 | 91 |
| 29.11 | Dimer | 232 | 1157 | [M-H]^-^ | 22 | 671 | 100 |  |  |
| 29.66 | Dimer | 232 | 1203 | [M+HCOO^-^]^-^ | 100 | 1157 | 100 | 671 | 19 |
| 29.66 | Dimer | 232 | 1157 | [M-H]^-^ | 40 | 671 | 100 |  |  |
| 29.91 | Dimer | 232 | 1203 | [M+HCOO^-^]^-^ | 100 | 1157 | 100 | 671 | 57 |
| 29.91 | Dimer | 232 | 1157 | [M-H]^-^ | 35 | 671 | 100 |  |  |
| 30.62 | Unknown | 228 | 593 | [M-H]^-^ | 100 | 285 | 100 | 473 | 20 |
| 30.62 | Unknown | 228 | 639 | [M+HCOO^-^]^-^ | 78 |  |  |  |  |
| 31.07 | Unknown | 232 | 867 | [M-H]^2-^ | 100 | 713 | 100 |  |  |
| 31.07 | Unknown | 232 | 890 | [M+Na]^2-^ | 64 | 867 | 100 |  |  |
| 31.94 | Dimer | 232 | 1157 | [M-H]^-^ | 100 | 849 | 100 | 1005 | 19 |
| 32.39 | Tetramer | 232 | 1180 | [M+Na]^2-^ | 100 | ND |  |  |  |
| 32.39 | Tetramer | 232 | 1157 | [M-H]^2-^ | 90 | ND |  |  |  |
| 33.76 | Unknown | 232 | 867 | [M-H]^2-^ | 100 | 713 | 100 |  |  |
| 33.76 | Unknown | 232 | 891 | [M+Na]^2-^ | 60 | 867 | 100 |  |  |
| 40.21 | Unknown | 232 | 913 | [M-H]^2-^ | 100 | 759 | 100 | 760 | 23 |

**Supplementary Table 5: Compounds detected with UHPLC-MS after incubation of gossypin with DyP2**. R.A= relative abundance.

| **RT** | **Tent. Annot.** | **UV_max_** | **m/z** | **Ion** | **R.A** | **MS^2^** | **R.A** | **MS^2^** | **R.A** |
| --- | --- | --- | --- | --- | --- | --- | --- | --- | --- |
| 2.22 | Unknown | 292 | 375 | [M-H]^-^ | 100 | 331 | 100 |  |  |
| 2.75 | Unknown | 224 | 347 | [M-H]^-^ | 100 | 329 | 100 | 184 | 39 |
| 4 | Unknown | 260 | 153 | [M-H]^-^ | 100 | 109 | 100 |  |  |
| 8.7 | Unknown | 232 | 853 | [M-H]^-^ | 100 | 809 | 100 | 673 | 45 |
| 8.7 | Unknown | 232 | 426 | [M-H]^2-^ | 35 | 401 | 100 | 329 | 87 |
| 8.7 | Unknown | 232 | 495 | [M-H]^-^ | 22 | 385 | 100 | 333 | 43 |
| 9.18 | Unknown | 296 | 495 | [M-H]^-^ | 100 | 385 | 100 | 333 | 49 |
| 10.03 | Unknown | 232 | 853 | [M-H]^-^ | 100 | 809 | 100 | 357 | 45 |
| 10.03 | Unknown | 232 | 426 | [M-H]^2-^ | 46 | 401 | 100 |  |  |
| 18.21 | Unknown | 232 | 631 | [M-H]^-^ | 100 | 477 | 100 |  |  |
| 18.21 | Unknown | 232 | 477 | [M-H]^-^ | 57 | 315 | 100 |  |  |
| 21.45 | Unknown | 228 | 509 | [M-H]^-^ | 100 | 333 | 100 | 399 | 31 |
| 21.45 | Unknown | 228 | 493 | [M-H]^-^ | 49 | 317 | 100 |  |  |
| 21.75 | Unknown | 232 | 631 | [M-H]^-^ | 100 | 477 | 100 |  |  |
| 21.75 | Unknown | 232 | 477 | [M-H]^-^ | 74 | 315 | 100 |  |  |
| 22.57 | Unknown | 232 | 269 | [M-H]^-^ | 100 | 269 | 100 | 241 | 22 |
| 23.14 | Gossypin | 228 | 479 | [M-H]^-^ | 100 | 317 | 100 |  |  |


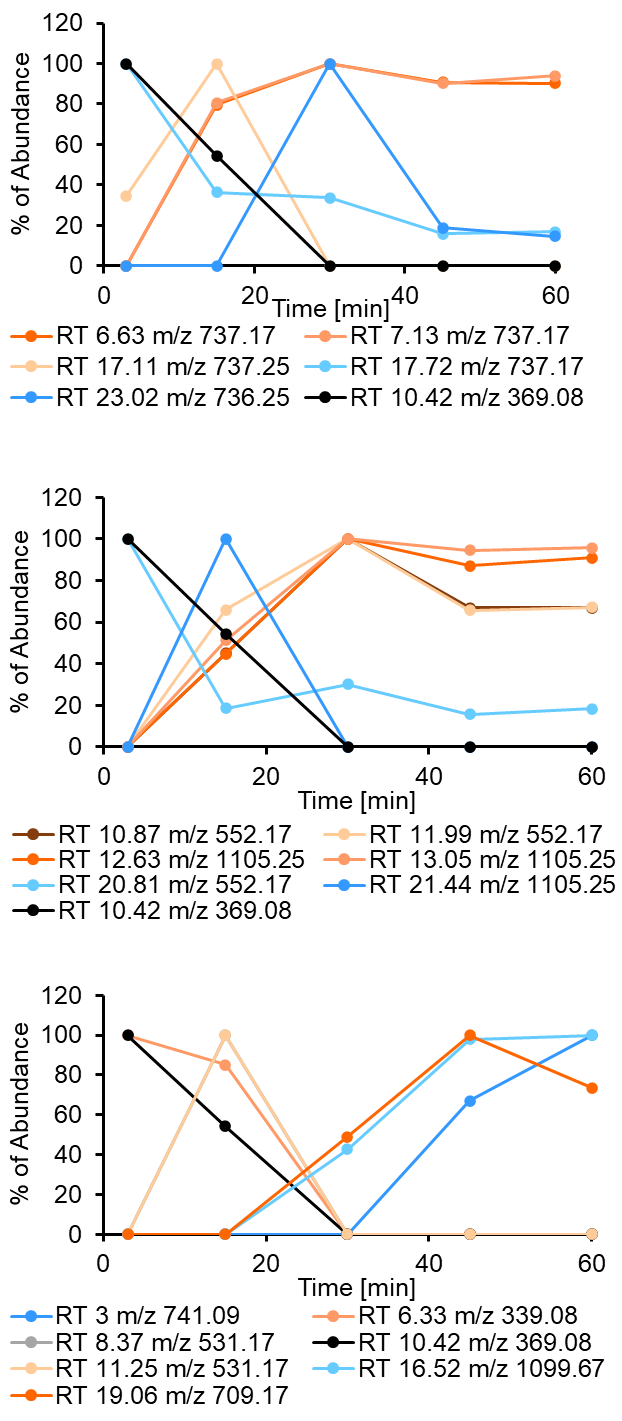


**Supplementary Figure 8:** **Time course of normalized MS peak areas of the fraxin reaction products.** The time point with the largest peak area was set to 100%. The incubations were conducted in duplicates but only one of the identical replicates is shown.


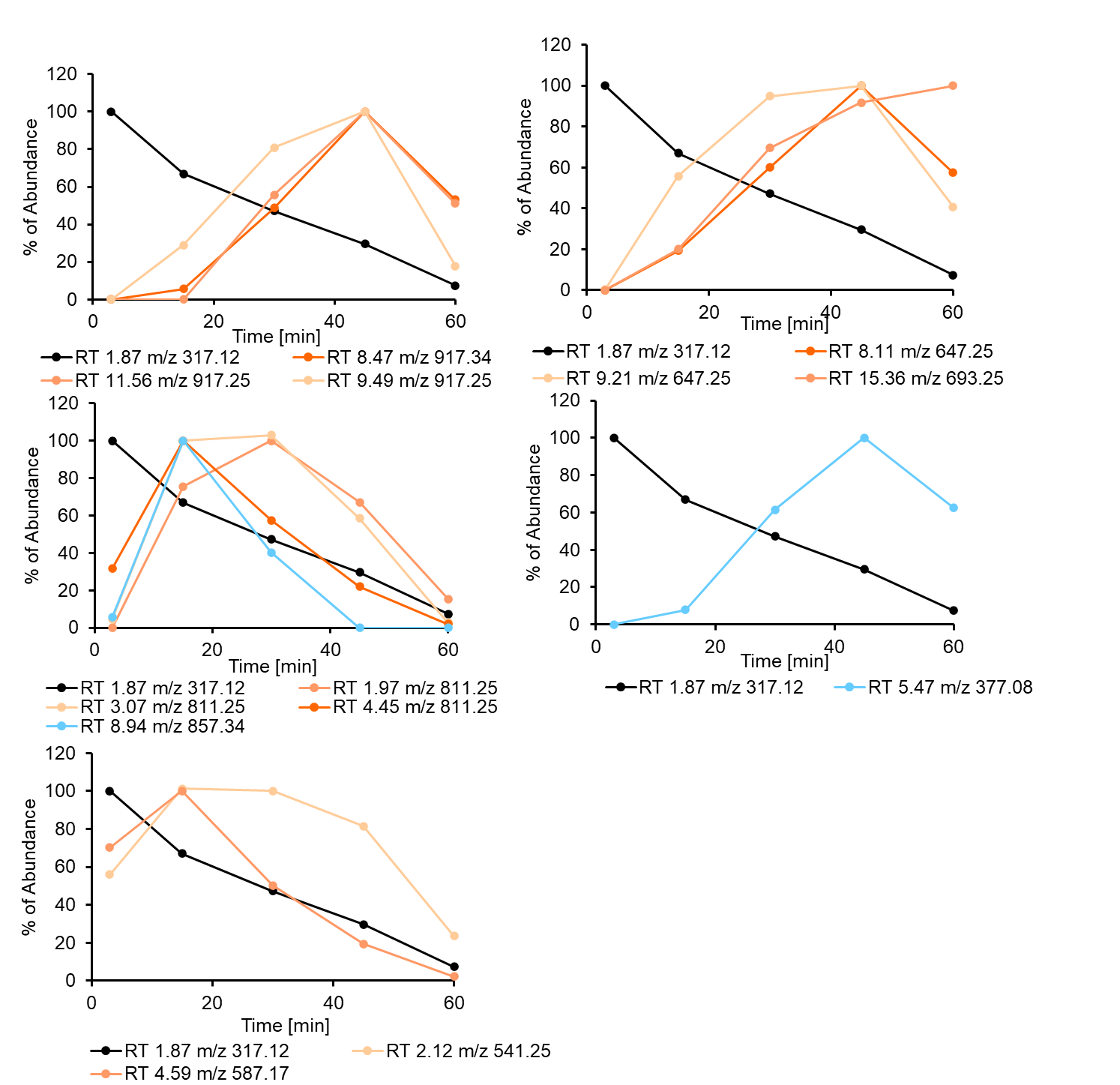


**Supplementary Figure 9: Time course of normalised MS peak areas of the arbutin reaction products.** The time point with the largest peak area was set to 100%. The incubations were conducted in duplicates but only one of the identical replicates is shown.


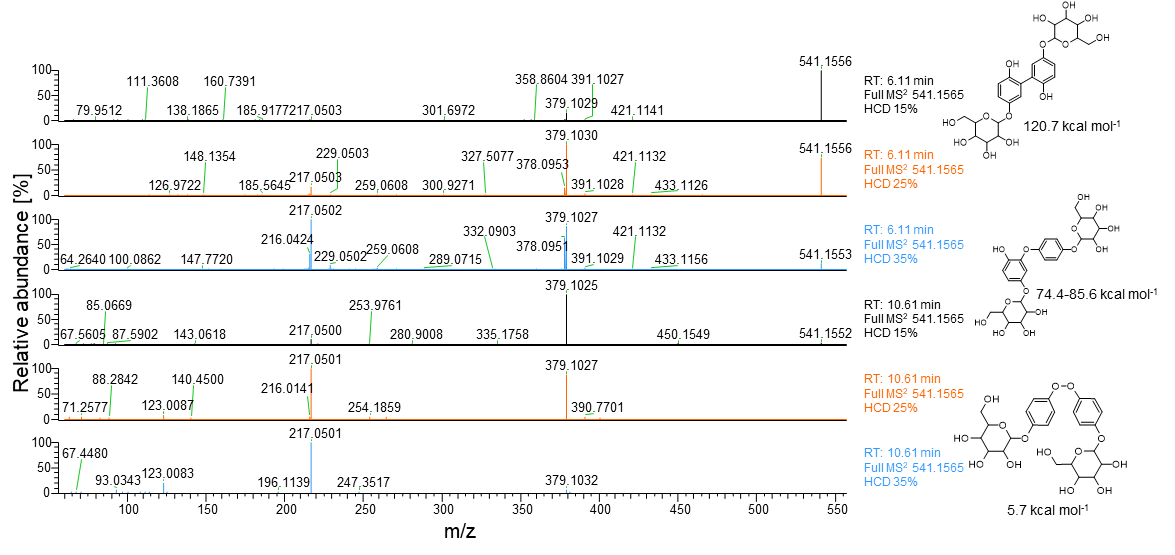


**Supplementary Figure 10: Comparison of HCD fragmentation energies in high resolution mass-spectrometry for arbutin dimers**. Arbutin 15-minute time point dimers were fragmented in HCD using 15, 25 and 35% collision energy. Dimer with RT 6.11 min needed high collision energy to fully fragment, whereas dimer with RT 10.61 min already was almost fully fragmented with the lowest collision energy. Hypothetical structures of dimers are presented on the right with predicted bond dissociation energies calculated with ALFABET (bde.ml.nrel.gov). Based on the HCD fragmentability, it is plausible that dimer at RT 6.11 min has C-C linkage, and dimer at RT 10.61 min has C-O linkage.


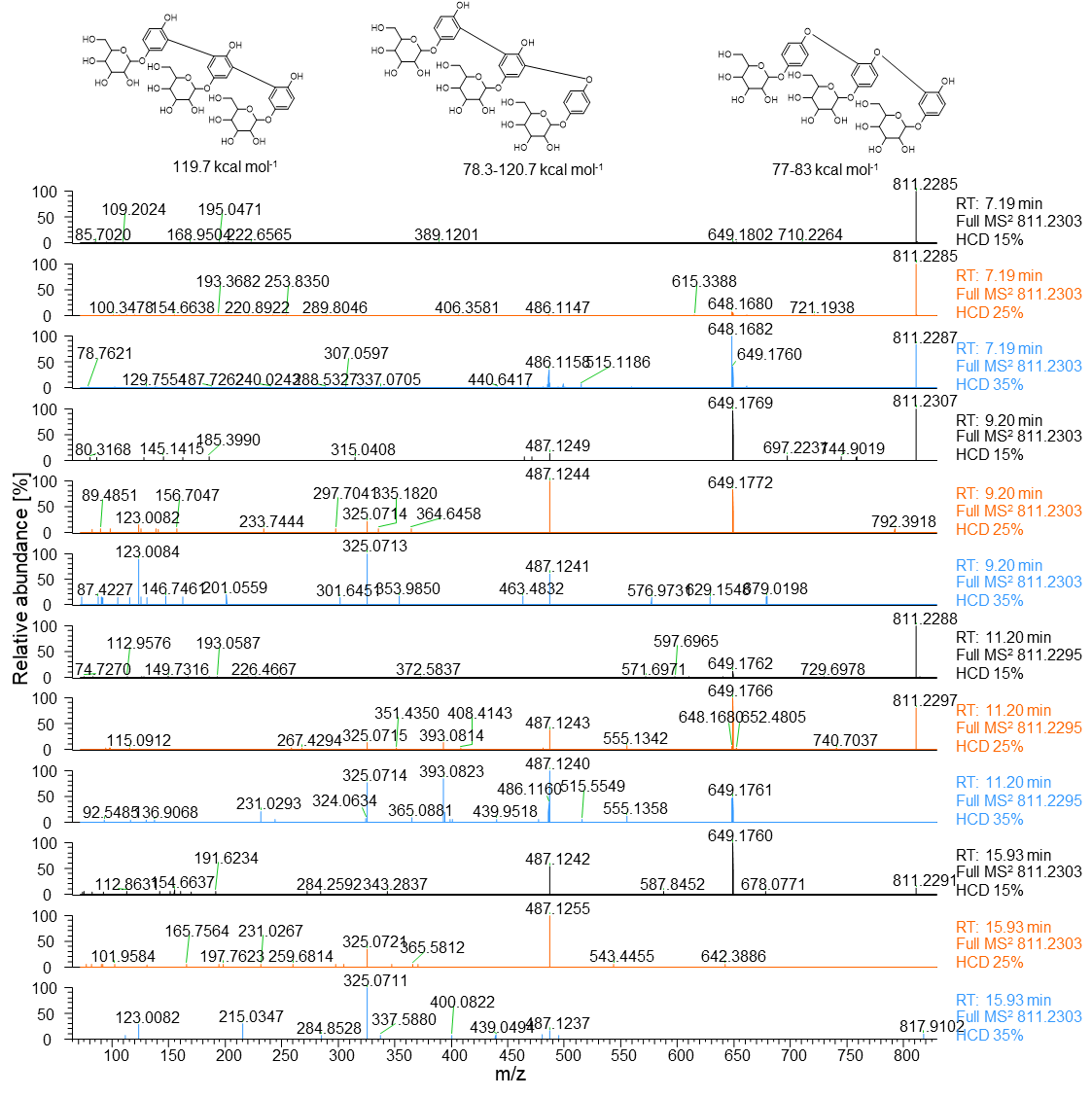


**Supplementary Figure 11:** **Comparison of HCD fragmentation energies of arbutin trimers in high resolution mass-spectrometry**. Arbutin 15-minute time point trimers were fragmented in HCD using 15, 25 and 35% collision energy. Trimers eluting at different times showed different fragmentation pattern with different collision energies. Hypothetical structures of trimers are presented on top of the figure with predicted bond dissociation energies calculated with ALFABET (bde.ml.nrel.gov).


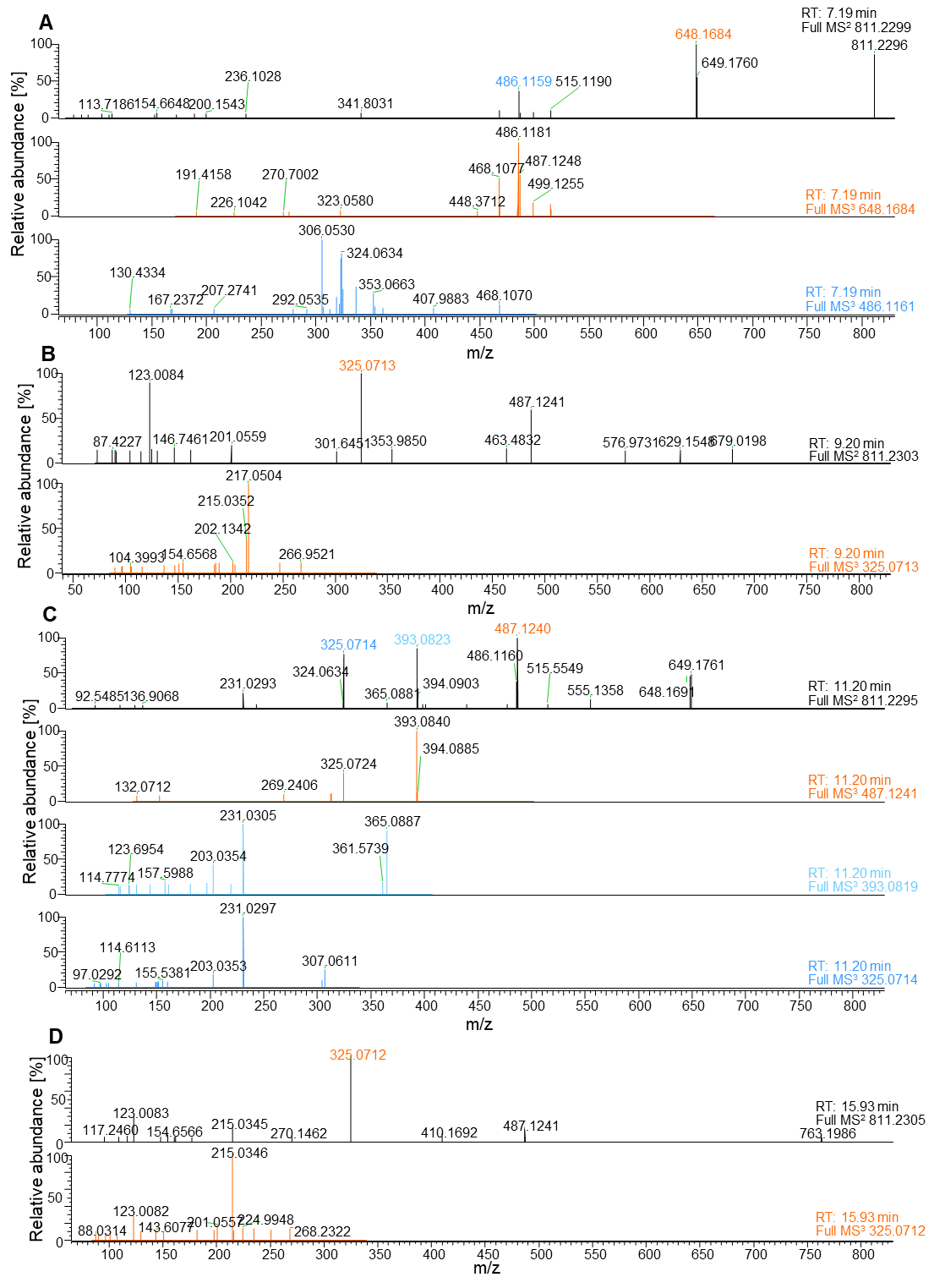


**Supplementary Figure 12: In-depth fragmentation of arbutin trimers.** Arbutin 15-minute incubation time point was analysed in high resolution MS IQ-X. By using acetonitrile gradient starting from 1%, the trimers eluted at **A)** 7.19 min, **B)** 9.20 min, **C)** 11.20 min and **D)** 15.93 min corresponded to the dimers at 1.97 min, 3.07 min, 4.45 min and 8.94 min in UHPLC-MS analysis starting at 5% acetonitrile. Full MS^2^ was obtained using HCD 35% collision energy and obtained MS^2^ fragments were further fragmented in MS^3^ using CID with 35% collision energy. Each trimer presents different fragmentation pattern indicating different linkages between arbutin monomers.

**Supplementary Table 6: Comparison of theoretical and measured masses in IQ-X.** Theoretical mass was calculated <https://www.sisweb.com/referenc/tools/exactmass.htm>

| **Chemical formula compound** | **Ion detected** | **Chemical formula ion** | **Exact mass** | **Mass detected** | **Mass difference (ppm)** |
| --- | --- | --- | --- | --- | --- |
| C24H30O14 | [M-H]^-^ | C24H29O14 | 541.1557 | 541.1569 | -2.217 |
| C18H20O9 | [M-H]^-^ | C18H19O9 | 379.1029 | 379.1029 | 0.000 |
| C12H10O4 | [M-H]^-^ | C12H9O4 | 217.0501 | 217.0503 | -0.921 |
| C6H4O3 | [M-H]^-^ | C6H3O3 | 123.0082 | 123.0084 | -1.626 |
| C12H8O3 | [M-H]^-^ | C12H7O3 | 199.0395 | 199.0398 | -1.507 |


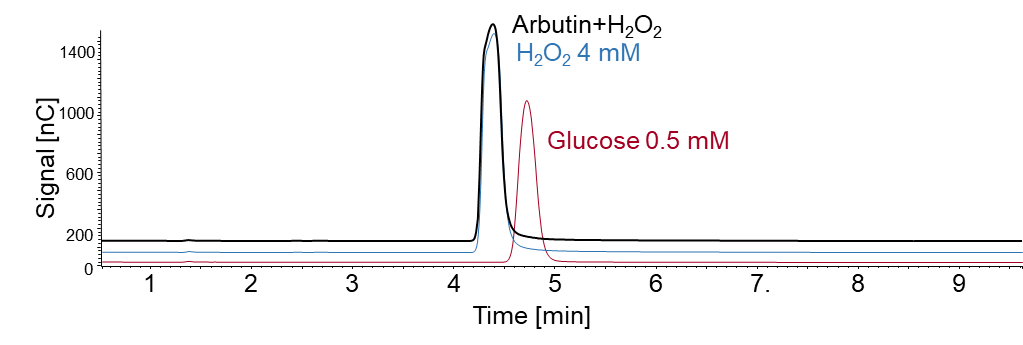


**Supplementary Figure 13:** **Elution of glucose and H_2_O_2_ in the used HPAEC method.** The peak at 4.3 min corresponded to H_2_O_2_ whereas the peak at 4.6 min corresponded to glucose.


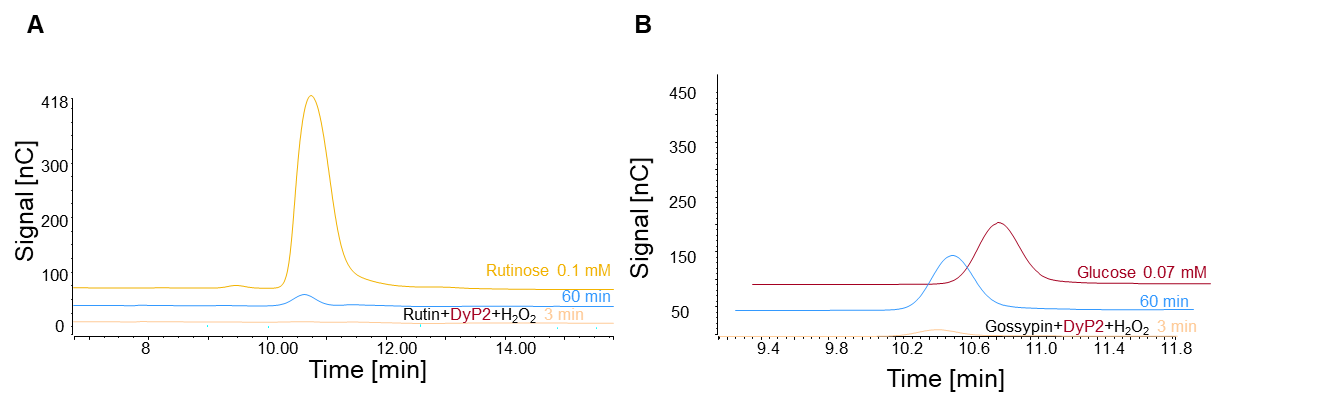


**Supplementary figure 14: HPAEC analysis of rutin and gossypin incubations with DyP2. A)** HPAEC chromatograms of rutin after 3- and 60-minute incubations with DyP2. Rutinose with defined concentration was used as a standard. The incubations were performed in duplicates but only one of the identical replicates is shown. Quantification of released rutinose from total substrate was not possible due to the solubility issue. **B)** HPAEC chromatograms of gossypin after 3- and 60-minute incubations with DyP2. Glucose with a defined concentration was used as a standard. The incubations were performed in duplicates but only one of the identical replicates is shown. Quantification of released glucose was not possible due to the solubility issue.


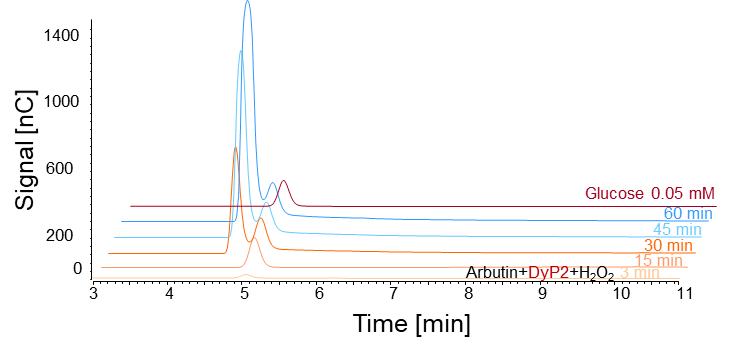


**Supplementary Figure 15:** **HPAEC time course chromatograms of released glucose after 0.26 mM arbutin incubations**. Glucose with defined concentrations were used as standards. The peak eluting at 4.4 min corresponds to unconsumed H_2_O_2_, and the peak eluting at 4.7 min corresponds to glucose. The incubations were performed in duplicates but only one of the identical replicates is shown. After 30 minutes H_2_O_2_ started to accumulate in the reactions whereas glucose concentration stayed the same.


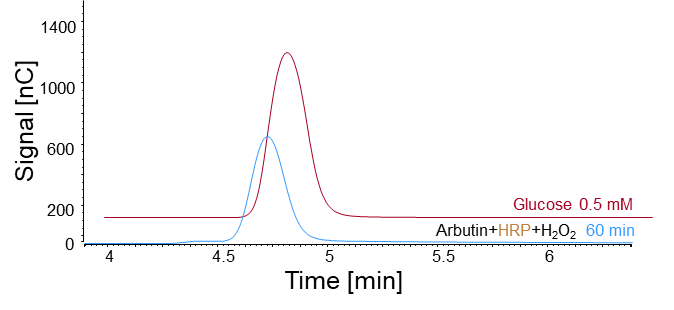


**Supplementary Figure 16: Glucose analysis of arbutin incubation with horse radish peroxidase (HRP).** Arbutin 2.6 mM was solubilised in 50 mM sodium acetate pH 4.5 buffer, 0.2 µM HRP was added, and the reaction was initiated with 200 µM H_2_O_2_. Fresh 200 µM H_2_O_2_ was added to the reaction after every three minutes for 60 minutes. Glucose with a defined concentration was used as a standard. Glucose was detected after 60 minutes.


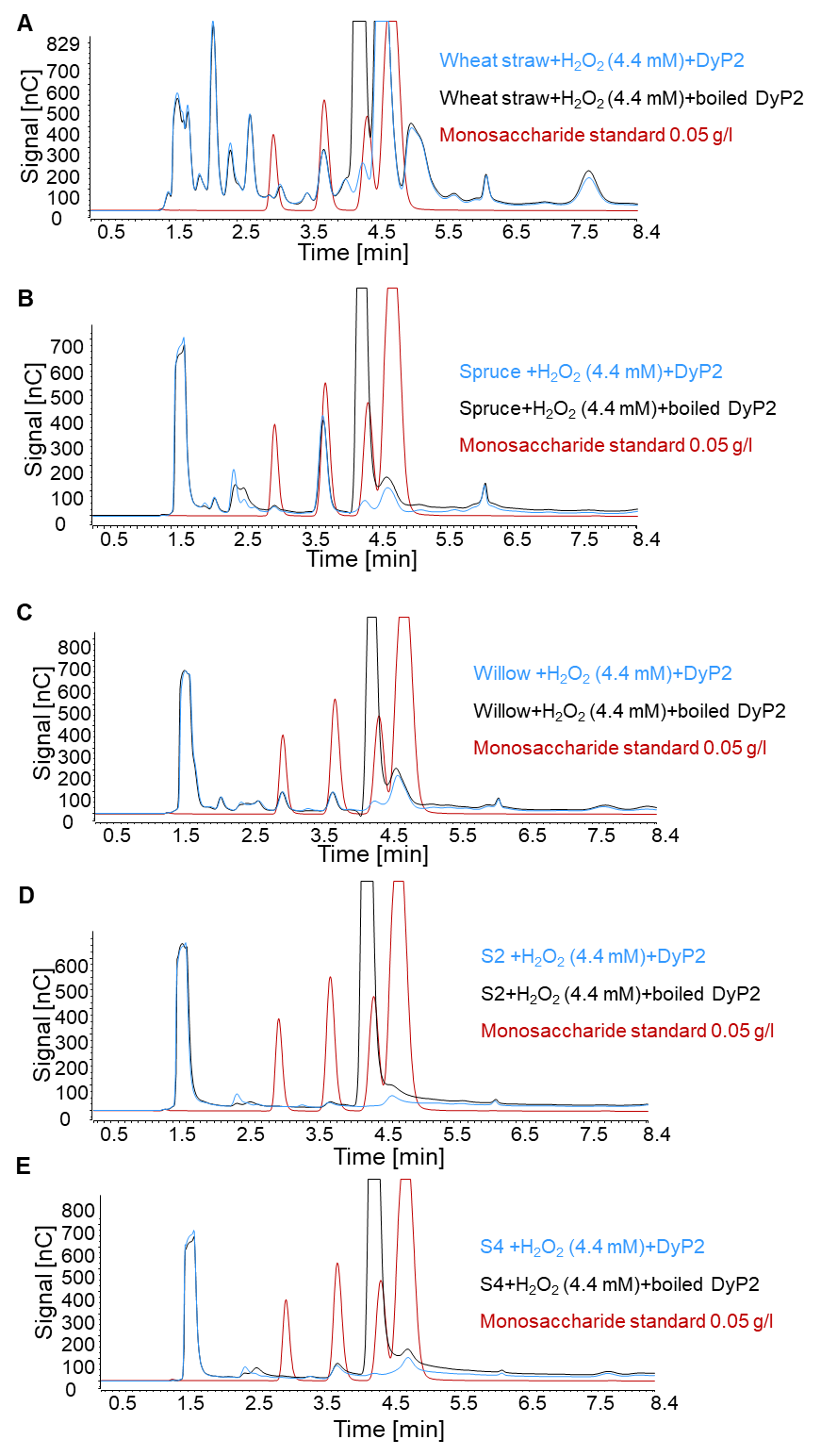


**Supplementary Figure 17: HPAEC analysis of lignocellulosic material.** Plant materials **A)** wheat straw, **B)** spruce, **C)** willow, **D)** water-soluble lignin fraction S2, **E)** water-soluble lignin fraction S4 were resuspended in 50 mM sodium acetate buffer pH 4.5, 2 µM enzyme was added, and the reaction was initiated with 200 µM H_2_O_2_. The reaction was continued by adding fresh H_2_O_2_ after every three minutes for 60 minutes. Substrates with H_2_O_2_ and boiled enzyme were used as negative control and monosaccharides were used as a standard. Incubations were conducted in duplicates but only one of the identical replicates is shown. New unknown peak at 2.5 min appeared in S2, S4, and spruce incubations.


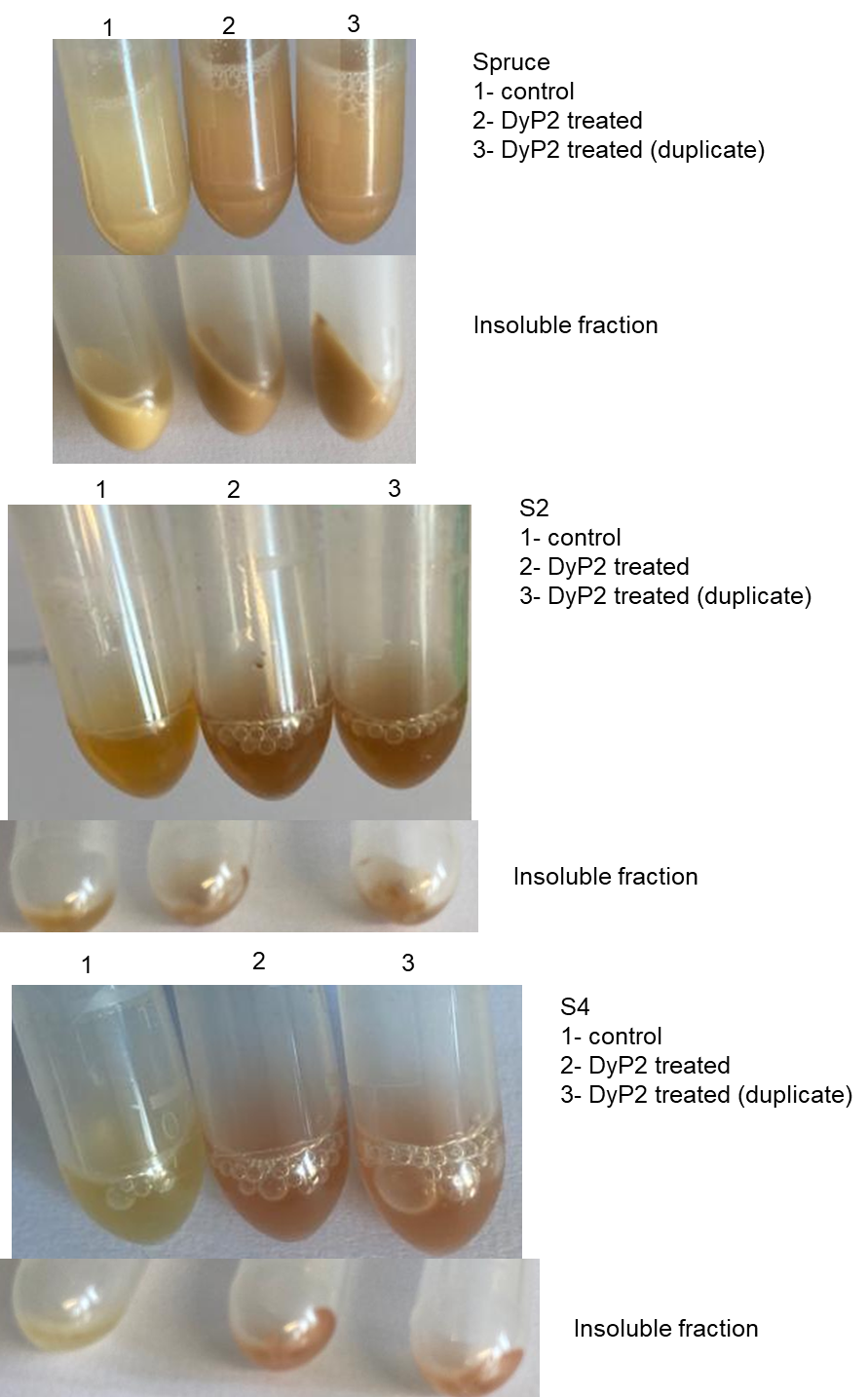


**Supplementary Figure 18: Pictures of spruce, water soluble lignin fractions S2 and S4 before and after enzymatic treatment**. S2 and S4 fragments were soluble in the buffer, but after DyP2 incubations precipitation occurred indicating oxidative polymerization. Spruce was partially soluble in buffer, but the colour changed from white to brown after enzyme treatment indicated polymerization. Substrate with H_2_O_2_ and boiled DyP2 was used as a control.

**Supplementary Table 7**: Pyrolysis-GC-HR-MS relative abundance of lignin-derived pyrolysis products in water-soluble lignin fractions in control and DyP incubations.

|  |  |  |  | **S2** | | **S4** | |
| --- | --- | --- | --- | --- | --- | --- | --- |
| **#** | **Compound** | **CAS** | **Structural feature** | **Control** | **DyP** | **Control** | **DyP** |
| 1 | phenol | 108952 | H, unsub. | 8.8 | 7.2 | 2.4 | 2.8 |
| 2 | guaiacol | 90051 | G, unsub. | 28.8 | 20.2 | 16.8 | 16.4 |
| 3 | 2-methylphenol | 95487 | H, methyl | 0.9 | 0.6 | 0.3 | 0.3 |
| 4 | 4-methylphenol (+3-MP) | 106445 | H, methyl | 1.2 | 0.9 | 0.4 | 0.4 |
| 5 | 4-methylguaiacol | 93516 | G, methyl | 1.3 | 1.0 | 0.6 | 0.7 |
| 6 | 2,4-dimethylphenol | 105679 | H, methyl | 0.6 | 0.3 | 0.2 | 0.1 |
| 7 | 4-ethylphenol | 123079 | H, misc. | 1.0 | 0.4 | 0.3 | 0.2 |
| 8 | 4-ethylguaiacol | 2785899 | G, misc. | 1.7 | 1.2 | 1.1 | 1.1 |
| 9 | 4-vinylguaiacol | 7786610 | G, vinyl | 28.4 | 30.6 | 53.9 | 39.1 |
| 10 | 4-vinylphenol | 2628173 | H, vinyl | 7.0 | 5.1 | 8.7 | 9.1 |
| 11 | eugenol | 97530 | G, misc. | 0.1 | 0.1 | 0.0 | 0.1 |
| 12 | 4-propylguaiacol | 2785877 | G, misc. | 0.4 | 0.2 | 0.2 | 0.2 |
| 13 | syringol | 91101 | S, unsub. | 12.4 | 12.4 | 7.0 | 9.8 |
| 14 | *cis*-isoeugenol | 97541 | G, misc. | 0.1 | 0.1 | 0.1 | 0.1 |
| 15 | *trans*-isoeugenol | 97541 | G, misc. | 0.6 | 0.6 | 0.3 | 0.4 |
| 16 | 4-methylsyringol | 6638057 | S, methyl | 0.5 | 0.6 | 0.3 | 0.5 |
| 17 | vanillin | 121335 | G, C_α_-O | 0.5 | 1.6 | 1.1 | 3.4 |
| 18 | homovanillin | 5603242 | G, C_β_-O | 0.1 | 0.3 | 0.1 | 0.3 |
| 19 | 4-ethylsyringol | 14059928 | S, misc. | 0.3 | 0.3 | 0.2 | 0.3 |
| 20 | acetovanillone | 498022 | G, C_α_-O | 0.3 | 0.8 | 0.3 | 0.6 |
| 21 | 4-hydroxybenzaldehyde | 123080 | H, C_α_-O | 0.0 | 0.1 | 0.1 | 0.3 |
| 22 | 4-vinylsyringol | 28343228 | S, vinyl | 2.4 | 5.7 | 3.2 | 5.0 |
| 23 | guaiacylacetone | 2503460 | G, C_β_-O | 0.6 | 0.8 | 0.4 | 0.6 |
| 24 | 4-allylsyringol | 6627889 | S, misc. | 0.1 | 0.1 | 0.0 | 0.1 |
| 25 | guaiacyl vinyl ketone | - | G, C_α_-O | 0.0 | 0.0 | 0.1 | 0.1 |
| 26 | guaiacyl diketone | 2034608 | G, C_α_-O, C_β_-O | 0.0 | 0.1 | 0.0 | 0.2 |
| 27 | cis-4-propenylsyringol | 26624135 | S, misc. | 0.1 | 0.1 | 0.1 | 0.1 |
| 28 | trans-4-propenylsyringol | 26624135 | S, misc. | 0.3 | 0.6 | 0.3 | 0.6 |
| 29 | dihydroconiferyl alcohol | 2305137 | S, C_γ_-O | 0.1 | 0.2 | 0.0 | 0.1 |
| 30 | syringaldehyde | 134963 | S, C_α_-O | 0.1 | 0.9 | 0.1 | 2.1 |
| 31 | cis-coniferyl alcohol | 458355 | G, C_γ_-O | 0.0 | 0.2 | 0.0 | 0.1 |
| 32 | homosyringaldehyde | - | S, C_β_-O | 0.0 | 0.2 | 0.1 | 0.2 |
| 33 | acetosyringone | 2478388 | S, C_α_-O | 0.4 | 1.1 | 0.2 | 0.6 |
| 34 | trans-coniferyl alcohol | 458355 | G, C_γ_-O | 0.2 | 2.0 | 0.2 | 1.3 |
| 35 | trans-coniferaldehyde | 458366 | G, C_γ_-O | 0.0 | 0.2 | 0.1 | 0.2 |
| 36 | syringylacetone | 19037582 | S, C_β_-O | 0.4 | 0.8 | 0.4 | 0.9 |
| 37 | syringyl diketone | 6925651 | S, C_α_-O, C_β_-O | 0.0 | 0.1 | 0.0 | 0.1 |
| 38 | dihydrosinapyl alcohol | 20736258 | G, C_γ_-O | 0.1 | 0.1 | 0.0 | 0.1 |
| 39 | cis-sinapyl alcohol | 537337 | S, C_γ_-O | 0.0 | 0.1 | 0.0 | 0.1 |
| 40 | trans-sinapyl alcohol | 537337 | S, C_γ_-O | 0.1 | 1.3 | 0.1 | 1.0 |
| 41 | trans-sinapaldehyde | 4206580 | S, C_γ_-O | 0.0 | 0.1 | 0.0 | 0.2 |
|  |  |  | Total C_α_-O | 1.4 | 4.8 | 2.0 | 7.4 |
